# Polymeric biomaterials-based tissue engineering for wound healing: a systemic review

**DOI:** 10.1093/burnst/tkac058

**Published:** 2023-02-07

**Authors:** Pratik Das, Suvendu Manna, Shivam Roy, Samit K Nandi, Piyali Basak

**Affiliations:** School of Bioscience and Engineering, Jadavpur University, 188, Raja Subodh Chandra Mallick Rd, Jadavpur, Kolkata 700032, West Bengal, India; Sustainability Cluster, University of Petroleum and Energy Studies, Dehradun 248007, Uttarakhand, India; School of Bioscience and Engineering, Jadavpur University, 188, Raja Subodh Chandra Mallick Rd, Jadavpur, Kolkata 700032, West Bengal, India; Department of Veterinary Surgery and Radiology, West Bengal University of Animal and Fishery Sciences, Belgachia, Kolkata 700037, West Bengal, India; School of Bioscience and Engineering, Jadavpur University, 188, Raja Subodh Chandra Mallick Rd, Jadavpur, Kolkata 700032, West Bengal, India

**Keywords:** Wound healing, Biomaterials, Tissue engineering, Skin regeneration, Polymers

## Abstract

**Background:**

Biomaterials are vital products used in clinical sectors as alternatives to several biological macromolecules for tissue engineering techniques owing to their numerous beneficial properties, including wound healing. The healing pattern generally depends upon the type of wounds, and restoration of the skin on damaged areas is greatly dependent on the depth and severity of the injury. The rate of wound healing relies on the type of biomaterials being incorporated for the fabrication of skin substitutes and their stability in *in vivo* conditions. In this review, a systematic literature search was performed on several databases to identify the most frequently used biomaterials for the development of successful wound healing agents against skin damage, along with their mechanisms of action.

**Method:**

The relevant research articles of the last 5 years were identified, analysed and reviewed in this paper. The meta-analysis was carried out using PRISMA and the search was conducted in major scientific databases. The research of the most recent 5 years, from 2017–2021 was taken into consideration. The collected research papers were inspected thoroughly for further analysis. Recent advances in the utilization of natural and synthetic biomaterials (alone/in combination) to speed up the regeneration rate of injured cells in skin wounds were summarised. Finally, 23 papers were critically reviewed and discussed.

**Results:**

In total, 2022 scholarly articles were retrieved from databases utilizing the aforementioned input methods. After eliminating duplicates and articles published before 2017, ~520 articles remained that were relevant to the topic at hand (biomaterials for wound healing) and could be evaluated for quality. Following different procedures, 23 publications were selected as best fitting for data extraction. Preferred Reporting Items for Systematic Reviews and Meta-Analyses for this review illustrates the selection criteria, such as exclusion and inclusion parameters. The 23 recent publications pointed to the use of both natural and synthetic polymers in wound healing applications. Information related to wound type and the mechanism of action has also been reviewed carefully. The selected publication showed that composites of natural and synthetic polymers were used extensively for both surgical and burn wounds. Extensive research revealed the effects of polymer-based biomaterials in wound healing and their recent advancement.

**Conclusions:**

The effects of biomaterials in wound healing are critically examined in this review. Different biomaterials have been tried to speed up the healing process, however, their success varies with the severity of the wound. However, some of the biomaterials raise questions when applied on a wide scale because of their scarcity, high transportation costs and processing challenges. Therefore, even if a biomaterial has good wound healing qualities, it may be technically unsuitable for use in actual medical scenarios. All of these restrictions have been examined closely in this review.

Highlights

- A detailed review of different polymeric biomaterials used for wound healing applications.
- Review on recent advances in skin substitutes and skin-based scaffolds.
- The clinical evidence for the use of different polymers and polymer composites in treating chronic wound healing is reviewed.
- Different drug delivery systems using polymeric biomaterials for enhanced wound healing are discussed.

## Background

Skin, the outer protecting layer, mostly prevents entry of pathogenic microbes, harmful radiation and toxic substances into body tissues. Hence it is one of the vital parts of the body’s defense mechanism. Wound healing consists of four phases: haemostasis, inflammation, proliferation and remodelling (Figure S1, see online supplementary material) [1]. However, loss of the skin’s self-healing capacity may lead to a major physiological imbalance, which can result in difficulty in wound healing conditions [2]. This can be altered by the application of skin and tissue engineering techniques [3,4]. Burn wounds, on the other hand, are highly complex wounds and require different approaches for treatment (Figure S2, see online supplementary material).

Biomedical engineering’s study of tissue and skin regeneration offers exciting possibilities for fixing or replacing malfunctioning organs and tissues (Figure S3, see online supplementary material) [5]. This technique can make use of specialized cell sources, biomaterials and an engineered extracellular matrix (ECM). Three categories can be used to describe synthetic skin substitutes [6].

- Depending on materials: biological, synthetic and biosynthetic.
- Depending on covering time: temporary and permanent.
- Depending on layer: epidermal, dermal and bi-layered skin substitutes.

**Figure 1:**
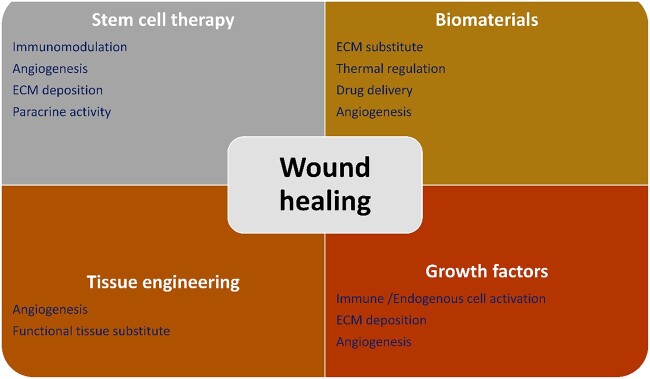
Different approaches for wound healing. *ECM* extracellular matrix

Several approaches have been adopted for the utilization of biomaterials as skin substitutes as they possess properties such as easy handling and application at the wound site, and sterility and are non-toxic and non-antigenic with minimal inflammatory reactivity [7–10]. They must be easily incorporated within the host with minimal pain, facilitating angiogenesis. Various approaches can be applied for effective wound healing, including stem cell therapy, the inclusion of growth factors and advanced skin tissue engineering (Figure 1).

The pivotal factors of the tissue engineering method include the type of biomaterials involved, the type of cells to be substituted, growth factors and signalling molecules along with engineering components like scaffolds [11–15]. Scaffolds, which can be made from either natural or synthetic biomaterials, play a pivotal role in the repair of damaged tissue by creating a new microenvironment that promotes cell survival, proliferation and differentiation. Many different biomaterials have been tried for treating chronic wounds [16,17]. A detailed description of the skin healing mechanism and tissue enginenring is provided in the supplementary file, see online supplementary material. However, some of the natural and synthetic biomaterials are reviewed in this article with their use in different types of wound treatment and the mechanisms involved in the healing process. Some of the most commonly used biomaterials are listed in Table 1.

Table 1 depicts the use of different natural and polymeric biomaterials for wound healing applications. The table provide information about the recent use and characteristics of synthetic polymers like polyurethane [18,19], poly caprolactone (PCL) [20,21], polyvinyl alcohol (PVA) [22,23], silicone [24,25] poly (lactide-co-glycolide) (PLGA) [26,27], poly-lactic acid (PLA) [28,29], poly ethylene glycol (PEG) [30,31] and natural polymers like collagen [32,33], chitin [34–36], chitosan [37,38], alginate [39,40], gellan [41,42], gelatin [43,44], fibrin [45,46], hyaluronic acid (HA) [47,48] and silk fibroin [49,50]. Each type of polymer is described in detail in the table along with one of its recent applications.

The current review inspects recent cases of the use of a wide range of biomaterials for wound healing purposes and provides a detailed insight into their mechanisms of action and effectiveness in wound healing.

## Methods

### Source

For the process of meta-analysis at the initial stage, three major research databases, namely SCOPUS, PubMed and Science Direct, were searched to collect related articles. However, almost all the articles found in the PubMed and Science Direct databases were available in the Scopus database as well, and therefore the SCOPUS database was used as the source for the collection of primary data for the review. The articles were selected from the database using relevant keywords and their combination via the following strings: string set 1: (‘biomaterial’ AND ‘wound healing’ AND ‘tissue engineering’); string set 2: (‘biomaterial’) AND (‘wound healing’ OR ‘tissue engineering’); string set 3: (‘biomaterial’ AND ‘tissue engineering’) OR (‘biomaterial’ AND ‘wound healing’).

This was undertaken to identify core themes of the research topic in terms of biomaterials for wound healing. In view of the core theme, the literature review was done for the most recent 5 years, from 2017–2021. The collected research papers were inspected thoroughly for further analysis.

### Data extraction and synthesis

After the identification of the core theme, the papers selected were subjected to extensive reading. To prevent bias in the selection of papers, a Preferred Reporting Items for Systematic Review and Meta-Analysis (PRISMA) flow chart was then prepared, demarcating the inclusion and exclusion criteria for the selected papers. Several bibliometric tendencies were gleaned from the SCOPUS database, and these were then used to evaluate the several different angles of study that have been undertaken with regard to the topic at hand.

**Table 1:** Properties and recent wound healing action of different polymeric biomaterials

**Types**	**Sl. No.**	**Name of the polymer**	**Details**	**Properties**	**Recent wound healing application**	**Reference**
Synthetic Biomaterials	1.	Polyurethane and its derivatives	• Organic homopolymer macromolecule linked together by carbamate (-O-CO-NH-) linkages • Thermosetting polymers or thermoplastic polyurethanes [18]	• Highly biocompatible and haemocompatible • High material strength and durability • Biodegradable with few exceptions • Highly elastic	A composite of polyurethane and gelatin was fabricated to act as an artificial skin scaffold for wound healing. Ciprofloxacin HCl was incorporated to avoid infection, thus promoting rapid wound healing. The electrospun scaffold was mechanically and thermally stable and pointed out to be a promising candidate for wound healing scaffold. *In-vitro* studies were conducted to evaluate the efficacy of the composite scaffolds.	[19]
	2.	Poly caprolactone	• Semicrystalline linear polyester • Partially crystalline • Synthesized by ring-opening polymerization of ε-caprolactone • The low melting point of ~60°C. Used in making specialized polyurethane that is resistant to water, oil, solvent and chlorine [20]	• Bio-degradable • Non-toxic • Semi-crystalline • Hydrophobic in nature • Slow degradation rate • Good elastic property	Electrospun nanofibrous scaffold composed of polycaprolactone and co-doped hydroxyapatite has been used as a material for wound healing. Aluminium/vanadate ions were incorporated for further modification. The scaffold was mechanically stable and contributed to cellular proliferation. The *in vitro* studies conducted using human fibroblasts indicated that the nanofibres could be a promising biomaterial for wound healing applications.	[21]
	3.	Polyvinyl alcohol	• Colourless, odourless, water-soluble synthetic polymer • Biodegradable, both in aerobic and anaerobic conditions • Synthesized using free-radical polymerization of vinyl acetate • Highly crystalline [22]	• pH-sensitive • film-forming capability • Water soluble • Bio-compatible • Hydrophilic	A very recent study showed the development of a polymeric nano-fibrous wound dressing of polyvinyl alcohol and propolis (a natural product with wound healing properties). The resulting scaffold exhibited excellent wound healing properties when tested on fibroblast cells and diabetic wounded mouse models. The scaffolds also supported tissue regeneration. The nano-scaffold turned out to be highly efficient in both *in vitro* and *in vivo* models using diabetes-induced male Swiss mice.	[23]
	4.	Silicone	• Inert synthetic compound • Colourless, oily or rubber-like polymers • Composed of siloxane. • Heat-resistant • Used in implants for tissue engineering • Soluble in non-polar solvents, including heptane, chloroform, toluene, benzene, etc. [24]	• Resistance to cold and heat • Good electrical conductivity • Good stability • Flexibility • Biocompatible	A novel multifunctional wound healing and dressing material was fabricated using poly(ε-caprolactone) quaternized silicone, outer layer and polyvinyl alcohol/collagen/quaternized chitosan, inner layer. The electrospinning technique was used for the fabrication. The membranes showed both antibacterial activity and cellular proliferation. The membranes exhibited excellent antibacterial activity, haemostatic performance, hydrophilicity, scar inhibition and wound healing properties. Experiments were conducted both *in vitro* and *in vivo*. The *in vivo* wound, healing and estimation of scar inhibition were evaluated using a rabbit ear full-thickness skin defect model.	[25]
	5.	Poly (lactide-co-glycolide) (PLGA)	• Co-polymer is composed of two different monomers, the cyclic dimers of glycolic acid and lactic acid • Food and Drug Administration (FDA) approved to be used in therapeutic devices • Glass transition temperature in the range of 40–60°C • Undergoes hydrolysis in the body • Soluble in solvents like chlorinated solvents, ethyl acetate tetra-hydro furan or acetone [26]	• Biocompatible • Biodegradable • FDA approved • Tuneable mechanical properties	Curcumin-loaded poly (PLGA) nanofibre membrane incorporated with growth factors (heparin) was fabricated for wound dressing as well as for the sustained release of the exogenous factor. The membrane was found to be highly biocompatible and also exhibited high tensile strength. The *in vivo* study showed fast wound closure, accelerated re-epithelisation rate, higher angiogenesis rate and more collagen deposition at the wound site. The nanofibrous scaffolds were tested using a streptozotocin-induced diabetic wound model in Sprague–Dawley rats.	[27]
	6.	Poly-lactic acid	• Aliphatic ester • Monomer connected with an ester bond • Naturally degradable • Used in various biomedical application • complex structures assembly like branched, star-shaped or grafted • Lacks mechanical toughness [28]	• Biocompatible • Non-toxic • Highly stable • Naturally biodegradable • Hydrophobic • Chemically inert • Tuneable mechanical property	Nanofibres consisting of polylactic acids were fabricated with the inclusion of black pepper essential oil or limonene and was coated with medium molecular weight chitosan. The fibres showed an enhanced anti-bacterial effect along with good mechanical properties. The fibres were found to be biocompatible and also promoted cellular adhesion and proliferation *in vitro*.	[29]
	7.	Poly ethylene glycol(PEG)	• Synthesized using low molecular weight polyether monomers derived from ethylene oxide • Repeating unit of ethylene glycol, −(O–CH2–CH2)– • Water-soluble polymer • Reported Tg and Tm are around −54°C and 74°C, respectively • Semicrystalline [30]	• Flexible • Non-toxic • Biocompatible	Injectable and degradable PEG hydrogel was synthesized using complex chemical routes with uniform pore size. The hydrogel showed superior biocompatibility with natural degrading capability. Both *in vitro* and *in vivo* results in the rat wound model showed that the injectable PEG could be considered to be a suitable candidate for wound healing applications.	[31]
Natural biomaterials	8.	Collagen	• Naturally occurring proteins are commonly found in the extracellular matrix of connective tissue • Consists of amino acids bound together to form a triple helix of elongated fibril [32]	• Biocompatible • Biodegradable • Strong • Permeable • Stable	A study showed the use of collagen and sodium alginate for the fabrication of a tissue scaffold to improve the effectiveness of stem cells in a full-thickness excision mice wound model. The scaffold was injectable, biodegradable and highly biocompatible with low immunogenicity. The scaffold loaded with human umbilical cord mesenchymal stem cells helps skin wound healing via partly inhibiting the NLRP3 pathway, hence a potential treatment for wound healing.	[33]
	9.	Chitin	• Hard naturally occurring polysaccharides, • long-chain polymer of *N*-acetylglucosamine [34] • Present in cell walls of fungi, the exoskeletons of arthropods, such as crustaceans and insects, the radulae of molluscs, cephalopod beaks, and the scales of fish and skin of lissamphibians [35]	• Translucent • Pliable • Resilient • Biocompatible • Biodegradable • Good tensile strength	Chitin, along with loose corn stalk and silver nanoparticles, was used to fabricate a sponge. The sponge was biocompatible and also exhibited a haemostatic effect when tested *in vivo* using a liver injury model of rats. The sponge also showed an excellent antibacterial effect and was also effective as a good wound closure agent when tested *in vivo* in male rats.	[36]
	10.	Chitosan	• Linear polysaccharide with randomly arranged • β-(1 → 4)-link • D-Glucosamine (deacetylated unit) and *N*-acetyl-D-glucosamine • Made from chitin shells of shrimp or dead crustaceans [37]	• Biodegradability • Non-toxic • Antifungal property • Can stimulate the immune system • Accelerates wound healing	Chitosan and nano-cellulose-based freeze-dried sponges were fabricated using EDC/NHS as a cross-linker. The sponges exhibited superior anti-bacterial efficacy owing to the inclusion of Lawson’s solution. The engineered scaffolds showed good wound healing activity as well as good haemostatic activity. The rat tail amputation model (amputation of tail caused due to wound) was used to check for the haemostatic the potential of the sample, and the rat full-thickness excisional cutaneous wound model were used to examine the wound healing assay.	[38]
	11.	Alginate	• Linearly arranged anionic biopolymer that is found in brown algae and bacteria • Consisting of α-l-guluronic acid (G) and β-d-mannuronic acid (M) residues arranged linearly in 1,4-glycosidic linkages • Biocompatible, biodegradable, low cost and readily available [39]	• Alginate is highly stable in both its solid and solution form • Water solubility • Viscous • High tensile strength • Flexible	A recent interesting study proposed the incorporation of *Raphanus sativus* L. Seed extracts in sodium alginate for wound healing application. The hydrogel was thermally and mechanically stable with proper angiogenic capability. The study pointed out the use of extract-loaded sodium alginate hydrogel as a potential candidate for wound healing application. The material was tested using the chick chorioallantoic membrane of fertilized chick eggs.	[40]
	12.	Gellan	• Linear, negatively charged polysaccharide. • Biodegradable and non-toxic • Poor mechanical strength • Needs blending with other naturally occurring polymers such as agar, chitosan, cellulose, sodium alginate, starch, pectin, polyaniline, pullulan, polyvinyl chloride and xanthan gum [41]	• Gellan is known for its stability, elasticity and ductility • Malleable • Biocompatible • Biodegradable • Flexibility	Recent research aimed at developing a novel bilayer wound dressing material using gellan gum and gelatin. Antibiotics were incorporated to increase the antimicrobial efficacy. The material showed good cellular proliferation when tested *in vitro*.	[42]
	13.	Gelatin	• Product of partial hydrolysis of collagen extracted from the skin, bones and connective tissues of animals • Gel-forming property • Translucent, colourless, flavourless [43]	• Good water-retention property • Dispersion stability • High dispersibility • Low viscosity	A composite of gelatin loaded with polysialic acid (a natural product with good wound healing properties) and crosslinked with tannic acid was constructed for wound dressing application with the ability to prevent bacterial infection. The fabricated material showed low toxicity and also exhibited a superior wound healing rate when tested using an excision wound model in rats.	[44]
	14.	Fibrin	• Natural fibres are formed when there is a healing injury in mammalian tissue. • Helps in blood clots and wound healing [45]	• Has both viscous and elastic properties • Withstands mechanical deformation • Biocompatible • Biodegradable	Liquid-type nonthermal atmospheric plasma (LTP) incorporated into a silk–fibrin composite gel was investigated for wound healing effect. The controlled release of LTP from the composite induced favorable cellular events in an irradiated wound bed. The different biological assays showed improved cellular viability and extracellular matrix deposition. *In-vivo* studies using full-thickness skin flap wounds on C57/BL6 mice also showed enhanced wound healing and regenerative properties.	[46]
	15.	Hyaluronic acid	• Naturally occurring chemicals are found abundantly in articular cartilage and synovial fluid • Non-protein-based glycosaminoglycan with characteristic physiochemical properties [47]	• Being one of the natural compounds of our body, it is highly biocompatible • Biodegradable • Clear/Translucent • Good fluid retention property	Combinational nanofibres consisting of hyaluronic acid/polyvinyl alcohol/polyethylene oxide blend and incorporated with ZnO NPs/cinnamon essential oil (CEO) antimicrobial combination exhibited both antimicrobial properties as well as superior wound healing. Physiochemical characterization and electron microscopy confirmed the presence of hyaluronic acid and ZnO NPs and CEO. The nanofibres inhibited the growth of *Staphylococcus aureus*. The *in vitro* and *in vivo* studies exhibited superior bio-compatibility and wound healing effects of the nanofibres.	[48]
	16.	Silk fibroin	• The major structural component of silk is produced by the silkworm • Composed of alternatively repeating units of hydrophobic heavy chains and hydrophilic light chains [49]	• Silk fibroin is known for its robust mechanical strength • High tensile strength • Stiff • Extensible • Flexible and malleable • Biodegradable and biocompatible	Silk fibroin-based scaffold incorporated with hyaluronic acid and natural silk fibroin nanofibres was constructed to serve as a novel scaffold for wound healing. The scaffold had high porosity (~92.5%), water-uptake ratio (~96%) and swelling ratio (~90%). The scaffold exhibited superior biocompatibility and cellular proliferation. *In-vivo* studies showed accelerated wound healing (up to 98.2 ± 0.5% within 4 weeks) and can also regulate collagen arrangement by nanofibres as a template to inhibit scar formation.	[50]

## Results

**Figure 2:**
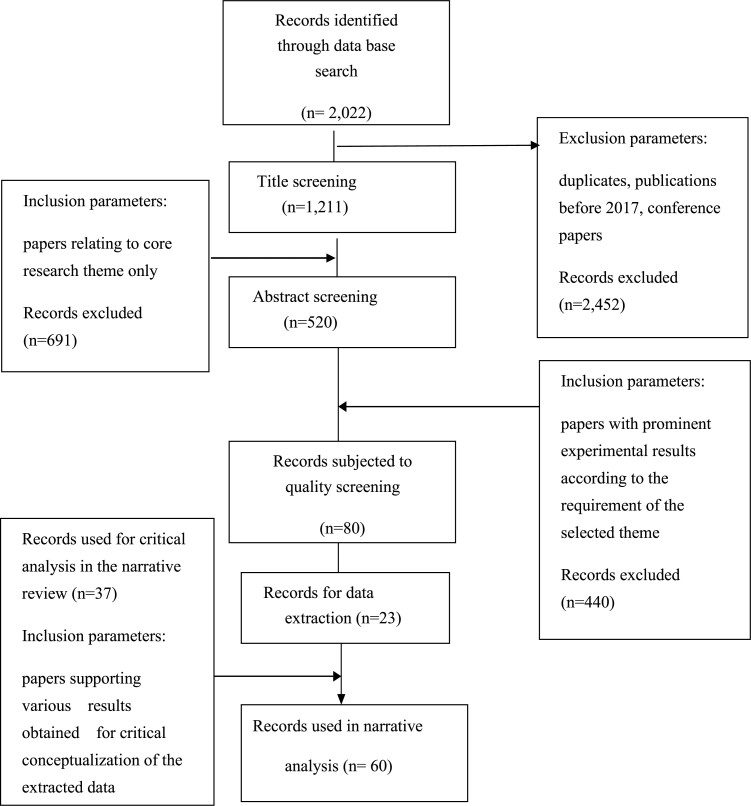
Preferred reporting items for systematic reviews and meta-analyses scheme for selection of articles for the systematic review

Database searches retrieved a total of 2022 research papers using the aforementioned keywords. After the removal of duplicates and papers published before 2017, ~520 were found to fit into the core theme (biomaterial for wound healing) and thus were finally subjected to quality screening. Quality screening considered parameters like relevance to the theme, aim of the study, methodology applied and results obtained. The papers excelling in all these parameters were taken forward for extensive analysis. Through this process, the 23 most suited papers were finally chosen for data extraction. PRISMA for the current review depicting the selection criteria, including exclusion and inclusion parameters for the current review can be seen in Figure 2.

For data extraction, the key data of the selected papers were screened and analysed on the following set of particulars: details of the authors with the year of publication, biomaterial type; classification; wound type; and used case (method of wound healing). These key data sets are summarized in Table 2 to aid further theme-wise analysis. From Table 2, it can be seen that both natural and synthetic polymers were used to prepare composites with wound healing capacities. In many cases, the researchers entrapped drugs or nanoparticles (with known antimicrobial properties) within the polymers and used them as scaffolds over the wound and found that they could accelerate the healing process [51] (Table 2). Some of the studies also used composites of natural chemicals and synthetic polymers to develop a hybrid hydrogel to be used in wound healing. The hybrid gels were found to enhance cellular growth in the wounded regions. However, those scaffolds were used to heal non-diabetic wounds [52]. This raises a potential problem with their efficacy in treating wounds in diabetic people. Recently, many researchers have tried to develop hybrid human cell–polymer composites and tested their potential in diabetic wound healing. Adipose-derived stem cells (ASCs), adipose-derived mesenchymal cells [53], human umbilical cord-derived cells and modified transforming growth factor (TGF)β3 and platelet-derived growth factor (PDGF) have been used for the preparation of such scaffolds by combining them with different natural or synthetic polymeric hydrogels [54]. Their reports showed fast proliferation of the wounded cells and fast repairing of wounded tissues even with no after-healing scar mark. However, all those studies have to be verified for their toxicity towards other cells, biocompatibility for non-host wounds and capability of suppressing localized inflammation before they are used for wound healing applications for both diabetic and non-diabetic patients.

**Table 2:** Analysis of recent approaches to fabricating wound healing construct using biomaterials (synthetic/natural)

**Sl. No.**	**Authors and year**	**Biomaterial type**	**Classification**	**Wound type**	**Used case (method)**	**Property of the material for enhanced wound healing**
1	Bienert *et al.* 2017 [55]	Native *B. Mori* silk membranes and growth factor-regulated silk membranes	Natural	A chronic wound in an *in vitro* model	All of the silk membranes were cytocompatible and exhibited enhanced chemokine (C-X-C motif) ligand 1 (CXCL-1) and monocyte chemo-attractant protein secretion. Epidermal growth factor (EGF) and vascular endothelial growth factor (VEGF)-regulated silk membranes accelerated macrophage adhesion. Dermal equivalents had higher wound healing capacity when covered with growth factor-regulated silk membranes.	• The silk membrane stimulated growth factors that were in direct contact with the membrane • Silk membranes stimulated macrophage release of neutrophils and thus inducing factors like CXCL-1 and MCP-1 while inhibiting the production of pro-inflammatory cytokines, including interleukin (IL)-6, IL-10 and TNF-α
2	Rossi *et al*. 2017 [56]	Chitosan ascorbate nanoparticles loaded with amoxicillin trihydrate	Synthetic and natural	Wound due to atrophic vaginitis in an *in vitro* model	Chitosan ascorbate NPs interact with mucin having an inhibitory effect against *Enterococcus hirae* and *Streptococcus pyogenes*. For faster wound healing, it stimulates the growth of fibroblasts.	• Chitosan ascorbate nanoparticles were found to be highly stable and were found to be effective as a drug-delivery polymer matrix • Chitosan-based polymeric nanoparticles showed superior cellular biocompatibility and enhanced cellular migration, thus achieving faster wound healing
3	Da Fonseca *et al.* 2017 [53]	Adipose tissue derived mesenchymal stem cells (MSC)	Natural	Excision wound in an *in-vivo* model (Male Wistar Rat)	MSC derived from Adipose tissue secreted extracellular vesicles, which enhanced the proliferation of fibroblast and keratinocytes, thereby leading to healing.	• Extracellular Vesicles derived from mesenchymal stem cells activate the ATK pathway in a miR-205-independent manner and hence can accelerate wound healing
4	Abdel-Mohsen *et al.* 2017 [51]	Hyaluronic acid/silver nanoparticles fibre	Synthetic	Diabetic wound in chronic ulcers using an *in vivo* rat model	*In situ* synthesis increased thermal stability and antimicrobial activity against *E. coli* and accelerated the healing process.	• The fibres showed enhanced crystallinity and thermal stability, which is required for wound dressing materials • The silver nanoparticles enhanced the antimicrobial property of the dressing • The presence of hyaluronic acid enhances the overall wound healing process as it promotes angiogenesis and also has an antioxidant effect
5	He *et al*. 2018 [57]	Curcumin/polyvinyl alcohol hydrogel	Synthetic	Skin and burn wound using an *in vivo* rat model.	The hydrogel with optimal Cur/PVA volume (1:5 at 20% Cur/PVA) exhibited antibacterial activity to *E. coli* and *S. aureus*. At a higher volume (1:10 at 10% Cur/PVA), it accelerated wound healing and reconstructed the epidermis during the 14th day of healing.	• Polyvinyl alcohol (PVA) promotes wound healing owing to its intrinsic properties like biocompatibility and biodegradability and mechanical strength [58] • PVA, when kept hydrated, maintains its structural ability, thus requiring fewer changes in dressing, and due to its transparency, wound healing can be monitored efficiently
6	Park *et al*. 2018 [59]	Phlorotannins and poly (vinyl alcohol) fabricated hydrogel	Synthetic	A chronic wound using an *in vitro* model.	PVA/PH hydrogel showed decreased tensile strength and tensile modulus, but the swelling ratio and ultimate strain showed a significant increase. On PVA/PH hydrogel, cell attachment and proliferation were increased on the first and fifth days compared to normal PVA hydrogel.	• Phlorotannins and poly (vinyl alcohol)-based hydrogel showed better cellular proliferation • The mechanical strength of the PVA-based hydrogel film played a key role in the wound healing process
7	Dhand *et al*. 2018 [60]	Collagen/quaternary ammonium silane (Coll-QOS) composite scaffolds	Natural and synthetic	Dermal wound using an *in vitro* model	Scaffolds were estimated for biocompatibility and cellular adhesion in dermal fibroblasts and fetal osteoblasts.	• Quaternary ammonium organosilane (QOS) crosslinked collagen nanofibres showed superior flexibility and thermal stability • The mats showed antibacterial activity, thus promoting effective wound healing • Collagen is chemotactic in nature and hence attracts fibroblast at the wound site • Collagen downregulates matrix metalloproteinase (MMPS) and hence promotes faster wound healing [61]
8	Yu *et al*. 2018 [62]	Cell sheet composed of adipose-derived stem cells (ASC)	Natural	Full thickness wound using *in vivo* model (nude mice)	Adipose-derived stem cell sheets enhance the healing process by decreasing scar formation and increasing the survival rate of wounded tissues within 28 days with fewer side effects.	• The adipose-derived stem cell sheets possess antifibrotic properties • The ASC sheets’ anti-scarring action was mediated in part by an increase in hepatocyte growth factor secretion • There was a considerable increase in C1q/TNF-related protein-3 produced by ASC sheets, which may be the cause of the reduced macrophage recruitment to the wound tissues • The paracrine elements included in ASC sheets help skin repair wounds more quickly and with less scarring. This makes ASC sheets an excellent choice for topical wound therapy
9	Ouyang *et al.* 2018 [52]	Chitosan-marine peptide hydrogels (CSMP)	Natural	Burn wounds using *in vivo* rabbit models	CSMP-treated wounds showed re-epithelialization and collagen fibre deposition on the seventh day and regeneration of epithelium on the 14th day, along with upregulating expression of FGF2 and VEGF.	• Chitosan possesses intrinsic antibacterial activity, thus promoting faster wound healing at the wound site • The hydrogel upregulates the expression of FGF2 and VEGF, which in turn can promote skin regeneration • Chitosan also reduces the inflammatory stage to start the onset of the proliferation stage [63]
10	Zhou *et al*. 2018 [64]	Sodium alginate-polyacrylamide based hydrogel crosslinked with novel divalent ion cross-linking (copper, zinc, strontium and calcium)	Synthetic	Full-thickness skin wounds in an *in vivo* rat model.	All divalent ions cross-linked hydrogels were analysed for swelling ratio, biocompatibility and antibacterial property with detection of VEGF and transforming growth factor beta (TGF-β) expression. Zinc cross-linked hydrogel showed a better response to antibacterial activities, cell viability, fibroblast migration, collagen deposition and formation of granulation tissue.	• The hydrogel crosslinked with zinc ions showed superior mechanical strength • An alginate gel may absorb wound fluid in the form of a dry powder, which can be used to keep the wound moist and reduce germs • Polyacrylamide, on the other hand, shows good skin tissue adherence and proper water absorption
11	Zhu *et al.* 2018 [65]	Aminoethyl methacrylate hyaluronic acid methoxy polyethylene glycol hybrid hydrogels and chlorhexidine diacetate (CHX)-loaded nanogels (Gel@CLN)	Synthetic	Skin wound at the dorsal position in a mouse model.	Gel@CLN showed swelling activity and low cytotoxicity with prolonged release of CHX. In addition to speeding up the healing process, its haemostasis abilities were quite prominent.	• Hyaluronic acid promotes wound healing by drawing fibroblasts to the wound site • HA also reduces inflammation and is responsible for the induction of the early proliferative phase [47] • Polyethylene glycol provides good mechanical support for wound dressing material • PEG also provides thermal stability to the hydrogel [30]
12	Liao *et al*. 2018 [66]	Hybrid alginate hydrogel crosslinked by calcium gluconate crystals deposited in PCL–PEG–PCL (PCEC) porous microspheres	Synthetic	Skin wound using an *in vivo* rat model	The PCEC microspheres/Alg hydrogel had compatibility and enhanced wound regeneration.	• PCL is highly viscoelastic with considerable mechanical properties making it highly suitable for wound healing applications • PCL is hydrophobic, so it is mostly blended with other materials • PCL’s high surface-to-volume ratio and porous structure improve homeostasis, cell migration and adhesion, wound exudate absorption, gas permeability for proper gas exchange, cellular migration, and proliferation [67] • PEG’s properties, like flexibility, hydrophilicity, biocompatibility and non-immunogenicity, make it highly useful for wound healing • PEG has good adhesive properties
13	Heydari *et al.* 2018 [68]	Novel poly (glycerol sebacate)/polyhydroxybutyrate (PGS/PHB) with simvastatin (SIM) and ciprofloxacin (CIP)	Synthetic	Skin wound	CIP loaded into the surrounding PHB part of the fibre showed burst release within 24 h that regulated wound infections. Similarly, SIM loaded to PGS core part lowered rate of release, therby providing time for wound healing.	• PGS exhibits biocompatibility and biodegradability, thus making it highly suitable for wound healing • PGS provides conducive surface for the cells to adhere to surface of the biomaterials [69]
14	Huang *et al.* 2018 [70]	Chitosan/hyaluronic acid hydrogels and PLGA microspheres	Synthetic	Skin wound	Chitosan/hyaluronic acid hydrogels and PLGA microspheres elevated angiogenic promoting and vascular endothelial growth factor secretion with higher antibacterial activity for wound healing.	• PLGA is a highly bio-degradable polymer; thus, it stimulates the rate of epithelization [30] • Chitosan possesses intrinsic antibacterial activity • Hyaluronic acid plays a major role in the faster completion of the inflammatory phase thus inducing the proliferative phase
15	Wang *et al*. 2019 [71]	Polypeptide-based FHE hydrogel (F127/OHA-EPL) with stimuli-responsive adipose-derived MSC exosomes	Natural	Chronic nonhealing diabetic wound	FHE@exosomes hydrogel enhanced proliferation, migration and tube formation activity in umbilical vein endothelial cells with the enhanced healing process for diabetic wounds with faster angiogenesis, re-epithelization and collagen deposition.	• Poly—L-lysine (EPL) is a natural cationic polypeptide derived from *Streptomyces albulus* that is biodegradable, antimicrobial and biocompatible [30] • Pluronic F-127 gel is mildly inflammatory and stimulates VEGF and TGF-1 [72]
16	Yang *et al.*, 2020 [73]	Human umbilical cord-derived MSC (hUCMSC)-derived exosomes (hUCMSC-exos) encapsulated in thermosensitive PF-127 hydrogel	Synthetic	Chronic refractory wounds due to diabetes mellitus	A combination of PF-127 and hUCMSC-exosomes showed a faster wound closure rate and increased expression of CD31 and Ki67 with the regeneration of granulation tissue. It also upregulated the expression of VEGF and TGFβ-1.	• At normal temperature, PF-127 hydrogel improves exosome survival • The PF-127 acted as an efficient delivery system for exosomes and hence promoted angiogenesis
17	Lu *et al.*, 2020 [74]	Enzyme crosslinked gelatin hydrogel with human adipose-derived stem cell (hASCs) spheroid	Synthetic	Murine Burn Wound	The hASCs recognized wounded cells and elevated growth factors secretion for angiogenesis and gelatin scaffold. It also enabled cell migration by fastening tissue regeneration on the 10^th^ day.	• Gelatin absorbs wound exudates and also keeps the wound site moist • Gelatin exhibits good attachment to fibroblast • The RGD peptide sequences found in gelatin help cells recognize their integrin receptors, which are essential for cell adhesion [75]
18	Xu *et al.* 2020 [54]	Modified TGFβ3 and platelet-derived growth factor (PDGF) combined with synthetic biomaterial.	Synthetic	Skin wound	TGFβ3 reduced scar formation through decreased myofibroblast proliferation with decreased collagen ratio. PDGF acted as a chemoattractant against macrophages and fibroblasts and increased the growth factor secretion for granulation tissue and matrix formation.	• PLGA is highly degradable and can efficiently deliver growth factors for faster wound healing • PLGA has tuneable and controllable mechanical properties making it highly suitable for wound dressing [30,54]
19	Ji *et al.* 2020 [76]	A hybrid system of PVA/gelatin hydrogel/frog egg-like microspheres	Synthetic	Skin wound	The release of *Rana chensinensis* skin peptides (RCSPs) elevated the healing process. H-FMS acted as a depot for the release of RCSPs over 9 days without burst release and showed biocompatibility.	• PVA increases the porosity and cell-matrix adhesion of the final material • PVA has mechanical properties mimicking that of skin [77] • Gelatin promotes cellular adhesion
20	Yu *et al.* 2020 [78]	Exosomes derived from bone marrow MSC pre-treated with atorvastatin (ATV)	Natural	Diabetic wound (full-thickness skin type)	Exosomes from ATV-pre-treated MSCs helped in diabetic skin defects treatment by increasing endothelial cell function through the AKT/eNOS pathway by upregulating miR-221-3p.	• Exosomes are highly efficient in cellular communication hence promoting cellular migration • MSCs induce angiogenesis • The MSC-derived exosomes were highly stable with low immune rejection
21	Zhao *et al*. 2020 [79]	Catechol–Fe^3+^ cross-linked poly (glycerol sebacate)-co-poly(ethylene glycol)-*g*-catechol prepolymer (PEGSD) and GTU (PEGSD-GTU) hydrogel	Synthetic	Full-thickness skin incisions	It showed rapid shape adaptation, regulated inflammatory response, accelerated collagen deposition and self-healing properties. As these hydrogels get dissolved or disintegrated in the application area, with time slight change in pH has been observed; the application of the material resulted in haemostasis of skin lesions with higher anti-bacterial activity against methicillin-resistant Staphylococcus aureus (MRSA).	• Poly-ethylene glycol (PEG) contributes highly to the viscoelasticity of the hydrogel and makes it suitable as an injectable material • Poly (glycerol sebacate) exhibits flexible mechanical properties making it highly suitable for wound dressing material • PGS is nonimmunogenic, noncytotoxic *in vitro*, and elicited a modest inflammatory response *in vivo* with negligible fibrous capsule development [80]
22	Wang *et al.* 2021 [81]	Novel 3D printable bionic hydrogels	Synthetic	Large, irregular-shaped wounds	CMC/PL hydrogels with glycidyl methacrylate-modified carboxymethyl cellulose and ε-polylysine showed higher compression modulus (238 kPa), stable rheological properties and microbial inhibitory effect. They increased VEGF and CD31 expression and accelerated granulation tissue regeneration.	• The carboxymethylcellulose dressing shields the wound site from external influences that may cause discomfort, increase infection or inhibit normal wound healing • The cellulose-derived polymer carboxymethylcellulose is non-toxic, non-allergenic, water-soluble and anionic, making it highly suitable for wound healing purposes • Carboxymethylcellulose possesses high biocompatibility and superior biodegradability and also mimics tissue structure[82]
23	Ji *et al.* 2021 [83]	Janus-structured nanofibres loaded with *R. chensinensis* skin peptides and silver nanoparticles	Synthetic	Skin wound	The Janus nanofibres lowered cytotoxicity and antibacterial activity and accelerated re-epithelialization, fibroblast proliferation and regeneration of capillaries and hair follicles within 14 days.	• Polyvinylpyrrolidone (PVP) used in the fibres is soluble in water and has good film-forming properties, thus making it highly suitable for wound dressing • PVP is biocompatible, non-toxic, non-ionic, inert, temperature-resistant and pH-stable

## Discussion

Wound healing is a complicated process involving myriad interactions within concerned cells. The study of wound healing includes the identification of damaged cells or tissues and finding biomaterials for accelerated healing methods with minimal scaring. The materials used as effective skin substitutes for accelerating the healing process must have properties like easy handling and application at the wound site, sterility, non-toxicity and non-antigenicity with minimal inflammatory reactivity along with biocompatibility, biodegradability and similarity to the ECM. Moreover, they should be pivotal in reepithelization and vascularization of the ECM.

In skin and tissue engineering, biomaterials play a pivotal role in enhancing the regenerative potency of human tissues, restoring damaged states along with re-establishing normal biological function. Biomaterials can be incorporated by using several fabrication techniques within the body, individually or in combination, to repair or replace the damaged tissues or as a provisional scaffolding material adopted to reconstruct desired tissues or organs. The main aim of their use relies on providing mechanical support for cell adhesion, proliferation, migration and differentiation of the generated tissue. Moreover, biomaterials act as temporary scaffolds that act exactly as ECM templates and are used in cell regeneration. Natural and/or synthetic biomaterials are widely studied in tissue engineering due to their biocompatibility and structural resemblance to native source tissue. Their application within *in vitro* biological models releases less toxic products upon degradation and enhances biological recognition for cell adhesion and function [5].

Several approaches have been adopted for the utilization of biomaterials. The artificial skin fabrication and tissue engineering methods used for the wound healing process include the type of biomaterials involved and the type of cells to be substituted and concern growth factors and signalling molecules along with the fabrication method [15]. Numerous biomaterials fabricated using polymers like gelatin, chitosan, PVA, PLA and polyurethanes have been used in several chronic wound treatments. With the advancement of scientific research, various different growth factors and biomolecules are now being incorporated to achieve effective wound healing in complex wounds like third-degree burn wounds. This paper, however, aims to review some of the natural and synthetic biomaterials, indicating the type of wound treatment and the mechanism involved in it.

The PRISMA analysis done in this work points to the recent advances in the use of synthetic and natural materials as wound healing agents. Table 2 discusses in depth the use of various materials, their prorperties and their wound healing capabilities. In brief, Table 2 discusses use of silk membranes for chronic wounds [55] and chitosan ascorbate-based nanoparticles for healing wounds due to atrophic vaginitis [56]. Apart from this, different hydrogels prepared with chemicals like curcumin/PVA [57,58], phlorotannins (PH) and PVA fabricated hydrogels [59], collagen/quaternary ammonium silane (Coll-QOS) composite scaffolds [60,61], cell sheets composed of ASCs [62], chitosan-marine peptide [63], novel divalent ion cross-linked sodium alginate-polyacrylamide-based [64], HA/methoxy polyethylene glycol hybrid hydrogels and chlorhexidine diacetate (CHX)-loaded nanogels [65], hybrid alginate crosslinked by calcium gluconate crystals deposited in poly(ɛ-caprolactone) (PCL)-PEG-PCL (PCEC) porous microspheres [66,67], poly (glycerol sebacate)/polyhydroxybutyrate (PGS/PHB) loaded with simvastatin (SIM) and ciprofloxacin (CIP) [68,69], chitosan/HA and PLGA microspheres [70], polypeptide-based FHE (F127/OHA-EPL) with stimuli-responsive adipose-derived mesenchymal stem cell (MSC) exosomes [71,72], human umbilical cord-derived MSC-derived exosomes (hUCMSC-exos) encapsulated in thermosensitive PF-127 [73], enzyme crosslinked gelatin with human ASCs (hASCs) spheroids [74,75], hybrid system of PVA/gelatin hydrogel/frog egg-like microspheres (HFMS) [76,77], exosomes derived from bone marrow MSC pre-treated with atorvastatin (ATV) [78], catechol–Fe^3+^ cross-linked PGS-co-poly(ethylene glycol)-g-catechol prepolymer (PEGSD) and synthon-modified gelatin (GTU) (PEGSD-GTU) [79,80], 3D printable bionic hydrogels [81,82] and Janus-structured nanofibrers loaded with *Rana chensinensis* skin peptides and silver nanoparticles (AgNPs) for skin wounds [83]. All these biomaterials have been used recently to treat several different types of wounds arising from burns, diabetes or other diseases.

Hydrogels are an effective type of biomaterial for wound healing in dressings due to their capacity to form protective barriers against pathogens along with the creation of a hydrated environment for a wound healing response [58]. Several researchers have used hydrogel for the fabrication process to enhance the rate of wound healing. In one study, an enzyme cross-linked gelatin hydrogel with hASC spheroids was used to accelerate the wound healing process. The hASCs recognized wounded cells, increased growth factor secretion for angiogenesis occurred, and by day 10 had facilitated cell migration and accelerated tissue regeneration [74]. Gelatin which was used as a base material for the study, facilitates cell adhesion and proliferation [84]. The RGD sequence in gelatin promotes cellular attachtment and thus heals wounds faster [75]. The study demonstrated that the use of hydrogel with cell spheroids was significantly more successful than other groups in promoting angiogenesis due to the cell spheroid’s ability to stimulate cell–cell signalling and vascular formation. The study pointed out that the application of the cell spheroids has significantly improved wound healing in a burn wound model using male Wistar rats [74]. Similarly, in another study, a combination of PF-127 and hUCMSC-exos resulted in a faster wound closure rate and increased expression of CD31 and Ki67, with the regeneration of granulation tissue. It enhanced the upregulation of vascular endothelial growth factor (VEGF) and TGFβ-1 expression [72,73]. Pluronic F-127 stimulates epidermal growth factor (EGF) and hence can be highly effective in wound healing [85]. Pluronic F-127 can also improve electroporation-induced permeability of cell membranes, hence avoiding necrosis [86]. PF-127 gels acted as an efficient delivery agent for this study delivering hUCMSC-exos to the full-thickness cutaneous wound in a diabetic rat model. The exosomes released by hUCMSCs have been found to promote angiogenesis and speed up the healing of skin wounds [87]. A novel hydrogel with glycidyl methacrylate-modified carboxymethyl cellulose (CMC) and ε-polylysine (PL) showed higher compression modulus (238 kPa), stable rheological properties and an inhibitory effect on bacteria, thus making it highly effective for wound healing applications. CMC is a highly biocompatible and biodegradable tissue-mimicking material [82]. The hydrogels increased VEGF and CD31 expression and accelerated granulation tissue regeneration [81]. Bacterial infections are the major cause for non-healing wounds [88], the CMC/PL hydrogels were highly effective in inhibiting bacterial infection (both gram positive and gram negative bacteria). The hydrogel was also effective in reducing reactive oxygen species (ROS) at the wound site which in turn promotes wound healing. The hydrogel was tested on a rat full-thickness infected skin defect model to evaluate the effect of biomaterials in wound sites with infections. An animal model study indicated that the hydrogel promoted granulation tissue growth, collagen deposition and revascularization [81].

PVA is a kind of biomaterial commonly used in the fabrication of hydrogels and is frequently used in wound healing applications [77]. This material possess both anti-protein fouling capabilities and biological inertness. The PVA hydrogels for wound healing mostly combine with other molecules to stimulate the healing response [57]. In a study, PH/PVA fabricated hydrogel exhibited decreased tensile strength and tensile modulus and had increased swelling ratio and ultimate strain. On PH/PVA, hydrogel cell attachment and proliferation were increased on the first and fifth days [59]. In another study, a hybrid system of HFMS was used for facilitating wound healing. Results showed that HFMS acted as a depot for the release of *R. chensinensis* skin peptides over 9 days, without burst release, and showed biocompatibility [76]. The scaffold fabricated here exhibited higher encapsulation efficiency thus ensuring a sustained release of *R. chensinensis* skin peptides at the wound site. The hybrid scaffold system showed good porosity and good compressive properties, thus making it mechanically suitable for wound healing applications. The *in vivo* experiments done on Wistar rats showed proper wound healing and also shortened the time period required for wound healing [76]. PVA scaffold with propolis nanoparticles was evaluated using murine NIH/3 T3 fibroblasts along with a diabetic non-contractile wound healing model. Results suggested a wound closure rate of 68% after 7 days [23]. PVA is highly appropriate as a biomaterial for wound healing because of its transparency, mechanical resilience, biocompatibility and biodegradability; it maintains a moist environment and, when cross-linked, it may remain in place for 4 days in the wound site [89,90]. As they maintain their structural integrity when hydrated, the properties of PVA hydrogels provide monitoring of healing with very few dressing changes [91]. PVA also ensures proper delivery of different bioactive agents and growth factors due to its excellent swelling property [92].

Hyaluronan or HA is a polysaccharide commonly used in hydrogels for wound healing. Hydrogels consisting of HA and chitosan have been used by several researchers to elevate angiogenesis and secretion of VEGF, with higher antibacterial activity, suggesting its potency as a wound healing therapeutic [70]. Furthermore, hydrogels containing HA also promote blood clotting and enhance antibacterial properties [93]. Similarly, chitosan has also been used to fabricate hydrogels with effective wound healing therapeutics [94]. *In situ* synthesis of HA/AgNP fibre increased thermal stability and antimicrobial activity against *Escherichia coli* and accelerated the healing process in both diabetic and non-diabetic rat models [51]. The polysaccharide HA is considered to be a versatile glycolsoaminoglycan with a wide variety of biological applications [95]. From the perspective of wound healing, HA plays a very important role and detailed research has pointed out its application in every stage of wound healing [96]. As the wound heals, it binds to fibrinogen, which initiates the clotting process, permitting the migration of inflammatory cells. By inducing swelling, it opens the door for cells to invade. By inhibiting the movement of neutrophils, it reduces inflammation. In the proliferative phase, it draws fibroblasts to the wound site, fills in gaps of newly formed ECM, creates cushioning and structural organization, stimulates matrix metalloproteinase (MMPs) for angiogenesis, and promotes keratinocyte migration and proliferation, and in the remodelling phase, it plays a vital role in normal and pathological scarring [97]. HA also exhibits anti-oxidant activity [98]. In recent years, its properties have been successfully incorporated into wound dressings in addition to its importance in the biology underlying the healing process of wounds, these include: Anika’s HYALOFILL, Anika’s HYALOMATRIX, Labs Genévrier’s IALUSET, and Contipro’s HYIODINE, which have all been investigated and proved to be useful in the treatment of chronic and burn wounds [97,99]. Chitosan ascorbate nanoparticles loaded with amoxicillin trihydrate interacted with mucin and showed an inhibitory effect against *Enterococcus hirae* and *Streptococcus pyogenes* and enhanced *in vitro* fibroblast proliferation for wound healing [56]. The research also focused on the development and optimization of an effective delivery matrix. In burn wounds created in a rabbit model treated with chitosan–marine peptides hydrogels, re-epithelialization and collagen fibre deposition were observed on the seventh day with epithelium regeneration by the 14th day and wound healing by the 21st day [52]. Chitosan/HA hydrogels and PLGA microspheres were used to elevate angiogenesis and to increase VEGF for wound healing and had an antibacterial activity, which also helped in tissue growth [70]. HA and carboxylated chitosan were mixed with human-like collagen with a transglutaminase crosslinker to form skin scaffolds. This human-like collagen/HA/carboxylated chitosan (GEL4) hydrogel was found to be biocompatible by enhancing the adhesion, proliferation and migration of L929 cells. In addition to this, it guards the wound against infection and bacteria that are dangerous from the outside [100]. Chitosan is a natural polymer that speeds up the healing process of wounds by activating inflammatory cells, macrophages and fibroblasts, hence enhancing the inflammatory phase of the wound healing process. As a result, the inflammatory stage of the wound healing process is shortened and the proliferation stage begins sooner [101]. Chitosan is a highly biocompatible natural carbohydrate-based polymer that has shown good haemostatic properties [102]. Experiments on 14 mixed-breed swine have shown that chitosan is superior to gauze in terms of haemostasis capabilities [63]. Pre-hospital haemorrhagic wound care may benefit from the use of chitosan dressings [102]. Chitosan is highly biodegradable [103] and is known to upregulate polymorphonuclear leukocytes and hence promotes accelerated wound healing. As a result, new tissue organization and granulation are improved [104].

However, many skin substitutes are also available for enhancing the wound healing process. Different types of skin constructs have been discovered by several researchers that can act as a substitute for ECM of the skin by mimicking it with the utilization of bio-molecules like collagen, HA etc. [105]. In a study, quaternary ammonium silane collagen nanofibres were electrospun by incorporating QOS and cross-linked *in situ* after exposure to ammonium carbonate. These scaffolds were evaluated for biocompatibility and cellular adhesion in dermal fibroblasts and fetal osteoblasts [60,61]. Collagen is the most prevalent protein in the body. Collagen’s function in wound healing is to recruit fibroblasts and promote collagen deposition in the wound bed [106,107]. New tissue growth is stimulated by collagen dressing technology, which also promotes autolytic debridement, angiogenesis, neovascularization and re-epithelialization [108–111]. Collagen, a fundamental component of the ECM, regulates the stages of wound healing [108]. Collagen I and IV fragments can be inflammatory mediators by serving as effective neutrophil chemoattractants, increasing phagocytosis and immunological responses and altering gene expression [112,113]. In addition, it has been proven that collagen plays a crucial role in creating an anti-inflammatory, pro-angiogenic wound macrophage phenotype via microRNA signalling [114]. To aid in the healing of a wound, the collagen I C-pro-peptide fragment attracts endothelial cells and thus triggers angiogenesis [115]. In addition to this, it is important to note that aberrant levels of collagen have been linked to the development of hypertrophic and keloid scars [116]. Collagen, owing to all its intrinsic properties, has been widely used as wound dressing material in both chronic and serious burn wounds [117–120].

It is well known that skin wound healing relies heavily on cellular migration and multiplication. MSC-derived extracellular vesicles (EVs) aid in wound healing by activating the AKT pathway in a miR-205-independent fashion. Intriguingly, putting a gel containing MSC EVs at the wound site in an animal model has been shown to hasten wound healing, which might pave the way for future research employing non-invasive models [53]. A2-P-mediated ASC sheets, on the other hand, improve neoskin quality and speed up wound healing. Directly inhibiting the development of fibroblasts into myofibroblasts is possible with HGF produced by ASC sheets. More importantly, ASC sheets generate increased amounts of C1q/TNF-related protein 3 (CTRP3), which can prevent macrophages from releasing CCL2 and so limit the recruitment of more macrophages into the wound tissue. The immunomodulatory and antifibrotic properties of the ASC sheets were greatly improved, and they also improved wound healing and reduced scarring [62].

PGS is another biomaterial that is widely used in wound healing and tissue engineering because of its stability and non-cytotoxic and non-immunogenic nature [69]. In a generalized manner, it can be formed by polycondensation of glycerol and sebacic acid [80]. Due to its wide applications in biomedical sectors for skin and wound healing [121], adipose, cartilage [122] and dental and bone applications [123], several novel fabrication techniques for the alteration or combination of PGS have been reported by several researchers. In one study, a biodegradable PGS/PHB was fabricated with SIM and CIP and was applied as wound healing material. As a result, the CIP loaded into the surrounding PHB part of the fibre showed burst release within 24 h that regulated wound infections. Similarly, SIM loaded to the PGS core lowered the rate of release, thereby providing time for wound healing [68]. In another study, an injectable catechol–Fe^3+^ cross-linked PGS-PEGSD-GTU hydrogel system was studied for the treatment of multidrug-resistant bacterial infection and full-thickness skin wounds. Fast shape-shifting, controlled inflammation, faster collagen deposition and spontaneous repair were among its observable traits. As these fabricated hydrogels are dissolved, a slight change in pH is observed, resulting in haemostasis of skin lesions with higher antibacterial activity against methicillin-resistant Staphylococcus aureus (MRSA) [79]. PGS is synthesized by a two-step polycondensation of glycerol and sebacic acid [124]. Both glycerol and sebacic acid are endogenous parts of the human body and are involved in various metabolic reactions [125]. PGS exhibits tailorable mechanical properties and is highly bio-degradable, thus making it highly suitable to be used as scaffold [80]. PGS can be made of composites with a wide variety of polymers like gelatin, PCL [67], poly(lactic co-glycolic acid), PLGA and poly(glycolic acid) for tissue engineering applications [80]. PGS has a good drug delivery capacity and hence antibiotics and growth factors could be loaded on it for delivery at the wound site [68]. PGS does not cause an immune response and it was shown to be noncytotoxic *in vitro* and to cause only a small amount of inflammation and fibrous capsule formation in living cells when tested *in vivo* [126,127]. Dialdehyde-terminated PEG (PEG–CHO) and adipic dihydrazide-modified alginate were developed, which provided wound healing activity using alginate hydrogels with enhanced gelation kinetics, shear performance. The hydrogel exhibited fast drug release at pH 7.4. Researchers also suggested that this self-healing alginate hydrogel is also excellent for drug delivery, such as in intestinal treatment using special drugs [128]. PEG-based materials are generally highly porous and PEG can also be made into a composite with a wide variety of polymers [31]. Biomedical applications benefit greatly from PEG-based materials that do not often elicit an immunological response in humans [129]. The biocompatibility and water solubility of this material allows it to be employed in the manufacture of hydrogels. PEG-hydrogels have been widely used for tissue regeneration as drug delivery matrices and cell transport vehicles [130]. With the correct formulation, PEG hydrogels can modulate a wide range of biological processes, from cell survival and proliferation to secretion and differentiation [131,132], thus making it highly attractive for wound healing. Injectable alginate-based porous microspheres incorporated with PCEC exhibited fibroblast attachment and proliferation and also showed improved wound healing activity when tested *in vivo* using a rat model [66]. Further studies could be made on the incorporation of various growth factors and natural biomolecules to reduce the initial inflammatory response. The material could be also crosslinked with natural crosslinkers like genipin to reduce further toxicity and inflammatory response.

However, it could be said that the heterogeneous nature of wounds, the patient’s pathological condition and the degree to which the wound has penetrated would suggest the complexity of the therapy utilized and its effect on the wound healing process [133]. The use of specific types of biomaterials depending upon these criteria would help in accelerated cell regeneration.

### Future perspectives

Basically, stem cells in skin tissue engineering have certain limitations, such as immune sensitivity, compromised survival rate and lower proliferation and differentiation rates. However, these complications can be ameleriorated by the application of biomaterials. Natural and/or synthetic biomaterials can be easily designed for various types of wounds for healing treatment according to their physical and biochemical attributes. However, the incorporation of stem cells along with biomaterials enhances the competence of repairing dysfunctional skin tissue and accelerates the wound healing process through improved epithelialization, granulation tissue formation and angiogenesis.

Biomaterials are also used to regulate the fate of stem cells after delivery by providing mechanical support. Hence increased clinical trials on the utilization of biomaterial should be encouraged to study their biochemical attributes for wound healing, tissue repair and regeneration. Efforts should be made for improved clinical analysis regarding the design and fabrication of biomaterials using various sophisticated techniques in order to develop easy-access and cost-effective techniques for wound healing and regeneration. A detailed discussion on future aspects of the recently reviewed biomaterials and a few limitations are discussed below.

Chitosan and its derivatives are some of the most promising biopolymers for wound healing. Due to their biocompatibility and biodegradability, the addition of active metabolites is being studied and implemented in present-day applications to obtain bioactive, biodegradable and sustainable wound dressing systems. Results are promising and show how different active metabolites can yield different outcomes and better promote wound healing, such as skin cell regeneration and tissue deposition. Nevertheless, poor stability and low shelf-life are major issues in clinical trials for chitosan-based biomaterials [134]; thus, further clinical trial studies are required [135].

Adipose tissue-derived mesenchymal cells (ADSCs) are highly biocompatible and can differentiate into multiple cell lineages. Due to their biocompatibility, they can intergrate themselves in different parts of the body, both in autologous and allogeneic settings. Recent studies have focused on the immunological aspects of the ASC lineage, with studies being conducted on the various subpopulation of ASCs with regard to their immunological profile [136]. ASCs can also be utilized to promote angiogenesis vascularization, modulate immune responses and induce epithelialization in the wound. However, very few studies have been done on the optimal time and routes of stem-cell administration and the optimum number of stem cells required to illustrate definitive effectiveness. Accordingly, additional research is needed in both clinical and pre-clinical settings [137].

Antimicrobial resistance is a growing concern with new emerging threats very often with ever-increasing antimicrobial resistance properties; thus, with the growing antimicrobial resistance pathogens, AgNPs come into play. AgNPS demonstrate huge potential when compared to conventional antibiotic drugs. Recent studies conducted are focused on HA acid coupled with AgNPs to treat and promote faster wound healing, especially in diabetic foot ulcer treatment application. In recent years AgNPs have gained increasing popularity for their medical applications in wound dressings and drug delivery [138]. Another threat in the field of microorganism management is biofilm. With complex procedures to manage biofilm, an easier alternative is required, and research is being conducted on silver nanotechnology. This offers a novel approach for improved wound healing [139].

Phlorotannins and PVA fabricated hydrogels also showed promising results, but further studies should focus on the *in vivo* pathways and how they can promote a better and faster repair mechanism. The pH-rich environment of the hydrogel suggests that it may be a promising candidate for a better wound dressing agent [59]. Collagen composite scaffolds should be studied for their future application in the medical field of tissue repair. Moreover, hybrid biomaterials with tuneable biodegradability specially tailored for each bio environment are the pivot of biomedical engineering and the future prospects of this approach to tissue engineering [60].

Biomaterials obtained from marine sources satisfy the need for an available source of raw materials; biocompatibility is a property that needs to be satisfied for clinical application. Biomaterials obtained from various sources of marine life [140] need to be further studied to validate their bio-acceptability. Hypersensitivity may lead to detrimental outcomes if not treated properly. Finding the more appropriate biomaterial for the right application could lead to a more advanced civilization.

The crosslinking of ions can greatly speed up the wound healing process as homeostasis is achieved by mimicking the environment found inside the human body [64], but further studies are required to evaluate safety and biocompatibility. As that study [64] pointed out that only zinc provided both satisfactory wound healing and biocompatibility and the other ions did not meet expectations, further studies need to be conducted on how to engineer the ions to get favourable results. Furthermore, the addition of slow-releasing antibiotics or phytochemicals can greatly improve such biomaterials in clinical application.

Aminoethyl methacrylate HA methoxy polyethylene glycol hybrid hydrogels and chlorhexidine diacetate (CHX)-loaded nanogels (Gel@CLN) showed great effects in a mouse model [65] and hence could be upscaled as a wound-healing agent. Also, other research is being carried out in primary human cells to evaluate its effect on human cell lines.

SIM loaded in the core of PGS fibres has shown good results in sustained drug release [68]. However, this is currently in the developmental stage. *In vitro* studies are being done, leaving a vast area to explore in animal studies and in achieving preclinical approval.

Chitosan/HA hydrogels and PLGA microspheres also exhibited good drug delivery [70] and proficient wound healing effects, although the current stage of development is dealing with the drug delivery aspect of the biomaterial; however, in doing so, the solubility is compromised. Ideally, the material should be able to release anti-microbial drugs in a controlled manner and dissolve when the release is complete. Further research is needed in clinical trials to evaluate its bio acceptability.

Polypeptide-based FHE hydrogel (F127/OHA-EPL) with stimuli-responsive adipose-derived MSC exosomes showed efficient wound healing in an animal model with controlled exosome release. The *in vivo* studies showed that diabetic full-thickness cutaneous wounds healed much faster, with faster angiogenesis, re-epithelization and collagen deposition at the wound site [71]. Further clinical trials could pave the way towards a new multifunctional hydrogel to be used in wound healing applications.

Enzyme crosslinked gelatin hydrogel with hASCs spheroids showed promising results [141], although all tests need to be performed according to international organization for standardization (ISO) standards and a human clinical trial is also highly recommended in the future in order to establish this material as a wound-healing agent. Non-healing wounds are becoming a major concern for the biomedical industry. PDGF has acted as a promising candidate to tackle wounds that are time consuming and hard to heal. An *in vivo* study showed positive results for wound healing in an animal model [54]. If upscaled to a human clinical trial, this is a promising biomaterial for wound healing. Silk-based membranes also showed promisitng results *in vitro* by regulating growth factors and promoting wound healing [55].

Exosomes derived from bone marrow MSCs pre-treated with ATV could treat complex diseases such as psoriasis, melanoma, dermatitis and burn wound damage repair [142], thus giving an indication of a biomaterial with multiple applications [78]. Proper testing with primary human cells and clinical trials could help the medical community to develop a multifunctional natural biomaterial for wound healing applications.

3D printing technology is the future of both the medical and engineering field. The introduction of 3D printing technology has allowed scientists to recreate biomaterials that would be very tricky/impossible to create with classical laboratory facilities. One such group of biomaterials are novel 3D printable bionic hydrogels, which showed promising results in an animal model with accelerated granulation tissue regeneration [143]. The benefits of 3D printed biomaterials are that they can be exactly matched according to the type of material required for the given application, making them more practical for use. However, rigorous testing needs to be done in order to establish 3D printed material in the medical market.

Janus-structured nanofibres loaded with *R. chensinensis* skin peptides and AgNPs would suit best for skin wound treatment; with good drug delivery and antibacterial effects, they hold promise for dermal and subdermal wound healing [83].

Although vigorous research is going on in the field of biomaterials and their application in wound healing, a few limitations exist that need to be addressed. After reviewing all recent works, a few notable limitations are as follows.

- For naturally derived polymers, even after the raw material is procured, transportation and storage are important factors to consider and are still a question which needs a specific answer. However, the use of sterile and portable containers and packages could be a solution to this present problem.
- Most of the studies conducted are laboratory based, with no indication for upscaling; thus, a huge gap is being created between research and product development. This problem could be addressed by more industrial collaboration and obtaining knowledge about the problems from the market point of view.
- Although ADSCs are highly bio-compatible, they become interconnected with the ECMs of the cell membrane, which is the first parameter limiting their clinical application. Their use is widespread in pre-clinical and clinical applications, however their failure to retain their integrity, even after allografting, has resulted in declining use in the clinical field. The problem of integrity could be minimized by the use of various natural filler materials like plant-based nanocomposites (jute or corn fibres), silk-based fibrins and polymers derived from natural sources.
- The toxicity of metal nanoparticles like AgNPs towards eukaryotic cells is one of the main side-effects of using AgNPs in clinical application. The toxicity of the AgNPs could be reduced and optimized by using green methods for their formulation or capping them with biocompatible, bioactive agents like phytochemicals and natural polymers.
- One of the main hurdles in crafting medical-grade biomaterials is the lack of research on the biocompatibility aspect. Due to their heterogeneous nature, biomaterials are prone to cause hypersensitivity reactions. The use of more natural polymers derived from bio sources and the use of food and drug administration (FDA)-approved polymers could eventually create a way to combat this problem.
- Major studies are conducted in either mouse or swine-based cell lines, whereas more emphasis should be given to conducting studies in primary dermal cell lines or human-based cell lines. Studies should be conducted on *in vitro* wound models created using dermal cell lines. Research using human dermal cells would provide more reliable data.
- Most of the biomaterials designed have applications limited to *in vivo* research lacking human trials. More clinical collaborations would help to design experiments for human trials and would also bridge the gap between preliminary laboratory research and clinical need.

Chronic wounds largely includes diabetic foot ulcers, critical burn wound, ulcers etc. Wound healing is complicated by a number of factors that must be managed in order to expedite the healing process while minimizing complications, treatment costs and patient inconvenience, including infection. Medical and nutritional optimization, surgical debridement, unloading (or compression), supervision of ischaemia, management and control of infection, and proper wound bed preparation and monitoring of different cytokines and growth factors are primary considerations. This review pointed to the use of different polymeric biomaterials from both natural and synthetic sources to treat critical wounds. The use of natural polymers like gelatin, collagen and chitosan showed considerable healing effects, although studies should be conducted on how different cytokines are involved in the healing effect and on the interaction of the polymers with different cytokines. The inability to heal from critical wounds is caused by a variety of underlying diseases that are specific to each chronic wound type. That is why it is crucial to know what is wrong with the wound. In order to improve current treatment techniques and healing rates, as well as to allow the development of new, more successful therapies, a greater understanding of the variations between the many types of chronic wounds at the molecular and cellular level is required. The current approach of using synthetic polymers like PGS and PLA provides stability and mechanical support to the wound, but at the same time, deep and detailed studies need to be performed on the effect of these polymers on the immunogenicity of the animal being treated. The biodegradability of such synthetic polymers should be tuned according to the wound requirement. After 4 weeks of routine care, wounds that have not healed properly should have the underlying pathology evaluated and potentially more advanced therapeutic agents should be tried for better results. However, choosing the right treatment is not always based on data. Standardization of such therapy is urgently required in clinical and experimental studies. The use of different medications during wound healing treatment should also be monitored closely, and special emphasis should be given to the side effects of the medication along with the effect of the medicine in the process of wound healing. The use of different naturally derived growth factors (decellularized plasma rich fibrin, plant-derived human recombinant growth factors and serum albumin) along with polymeric scaffolds could enhance the wound healing duration with no genotoxic effect. On the other hand, pathogen infections continue to be a major issue and a major challenge for medical professionals. Appropriate treatment can fail, resulting in the recurrence of the disease or its spread to new tissues or even to other organs. In this day and age of rising medication resistance, treating pre-existing infectious problems is becoming increasingly challenging. The use of different new natural antibacterial and antimicrobial agents could bring a solution to this problem. Different plant-based extracts and phytochemicals like phenols and tannins have shown their efficacy as antibacterial materials [144] and could be loaded into polymeric scaffolds for antibacterial effect at the implantation site. There is a long way to go in terms of improving care for chronic and burn wounds. Tissue-engineered skin substitutes, stem cell therapy with MSCs, and existing polymeric dressing with mats and films have all provided impressive insight for better treatment, but at the same time, the scientific community should continue to investigate various ways to identify unexplored avenues of treatment and new targets.

## Conclusions

The above review presents a critical picture of the impact of biomaterials used in wound healing processes. Depending on the severity of the wound, different biomaterials have been tried to speed up the healing process. However, there are some ambiguities in the use of some of the biomaterials on a large scale due to their lack of availability, huge transportation costs and processing difficulties. This means that even a biomaterial with promising wound healing qualities may be technically unfit for use in clinical settings. In this review, all such limitations have been critically reviewed. In many studies, the wound healing potential of nanoparticle-containing biomaterials, nanofibres, 3D printed membranes and bio-inspired materials have been investigated in *in vitro* studies. Such biomaterials and their composites should have also been tested *in-vivo* for a better understanding of their potential in actual cases; before a biomaterial could be used for any wound healing, it should be thoroughly tested for toxicity, hypersensitivity, tissue-specific integrity, changes of physicochemical properties and upscaling possibilities in both *in vitro* and *in vivo* studies. All this information would guide medical practitioners in choosing the right biomaterials for specific types of wound healing applications.

## Supplementary Material

Suppliment_Intro_tkac058

## Abbreviations

CIP: Ciprofloxacin; CMC: Carboxymethyl cellulose; ECM: Extracellular matrix; GTU: Synthon modified gelatin; HFMS: PVA/gelatin hydrogel/frog egg-like microspheres; hUCMSCs: Human umbilical cord mesenchymal stem cells; LTP: Liquid-type nonthermal atmospheric plasma; PCL: Polycaprolactone; PDGF: Platelet-derived growth factor; PEG: Poly-ethylene glycol; PL: ε-Polylysine; PLGA: Poly (lactide-co-glycolide); PVA: Polyvinyl alcohol; SIM: Simvastatin; TGFβ-1: Transforming growth factor beta-1; VEGF: Vascular endothelial growth factor.
