# Supplementary material for: Polymeric biomaterials-based tissue engineering for wound healing: a systemic review
Source: Burns Trauma. 2023 Feb 7;11:tkac058. doi: 10.1093/burnst/tkac058 (PMC9904183; doi:10.1093/burnst/tkac058)
Supplement: Suppliment_Intro_tkac058 [file suppliment_intro_tkac058.docx]

**INTRODUCTION**

Tissue engineering mechanism corresponding to skin regeneration is getting maximum popularization in the field of wound healing treatment with the introduction of more novel fabrication techniques that use natural and/or synthetic polymers and stem cells or both [1]. The skin is a dynamic, continuously renewing organ that is divided into three major layers: the epidermis, dermis, and subcutis. The epidermis is the skin's outermost layer, and the subcutis is its innermost layer, with the dermis in between. The epidermis is made up mostly of keratinocytes and is divided into layers based on keratinocyte maturation [2]. The chemical barrier of the skin stands significant because the skin is naturally inhabited by a wide variety of microorganisms such as bacteria, fungi, and viruses. The chemical barrier acts through pH modulation and host defense peptides (HDPs), pH regulation being a necessary event for the activation of numerous enzymes and the inhibition of bacterial growth [3]. Due to their anti-pathogenic effect, HDPs are produced through the skin as one of the first lines of defense [4]. Keratinocytes in the skin produce chemokines, cytokines, and HDPs like MCP-112, tumor necrosis factor (TNF), IL-6, IL-8, and LL-3714. These molecules are an integral part of the innate immune response that recruits leukocytes, neutrophils, and monocytes. When a breach forms in the skin, these molecules are critical for initiating the healing process and, eventually, preventing fluid loss and invasion of opportunistic microorganisms through the wound by repairing the breach [5]. This complexity of skin structure makes it particularly hard for rapid regeneration in unfavorable or synthetic conditions. Hence, any small or large disruptions on the skin integrity lead to its disorientation, followed by tissue disintegrations that result in acute or chronic wounds [6,7]. Both acute and chronic wound formation and development patterns are different from each other, and the healing process for both of them also varies. Acute wounds mostly include 1st-degree burns or surgically created wounds or wounds by minor accidents, which heal within less period, whereas chronic wounds include diabetic, venous, or post-surgical wounds and have prolonged healing steps involved [8].

The wound healing process involves several steps, including homeostasis, blood clotting, inflammation, cellular proliferation/matrix deposition, and remodelling[9] [Figure S1], and it also involves the secretion of several growth factors [10,11]. However, the healing pattern varies greatly depending upon the type of wound. In the case of complex burn wounds (2^nd^ Degree or 3^rd^-degree burns), both epidermis and dermis are mostly damaged; hence the repair process is prolonged and even more complicated [12].


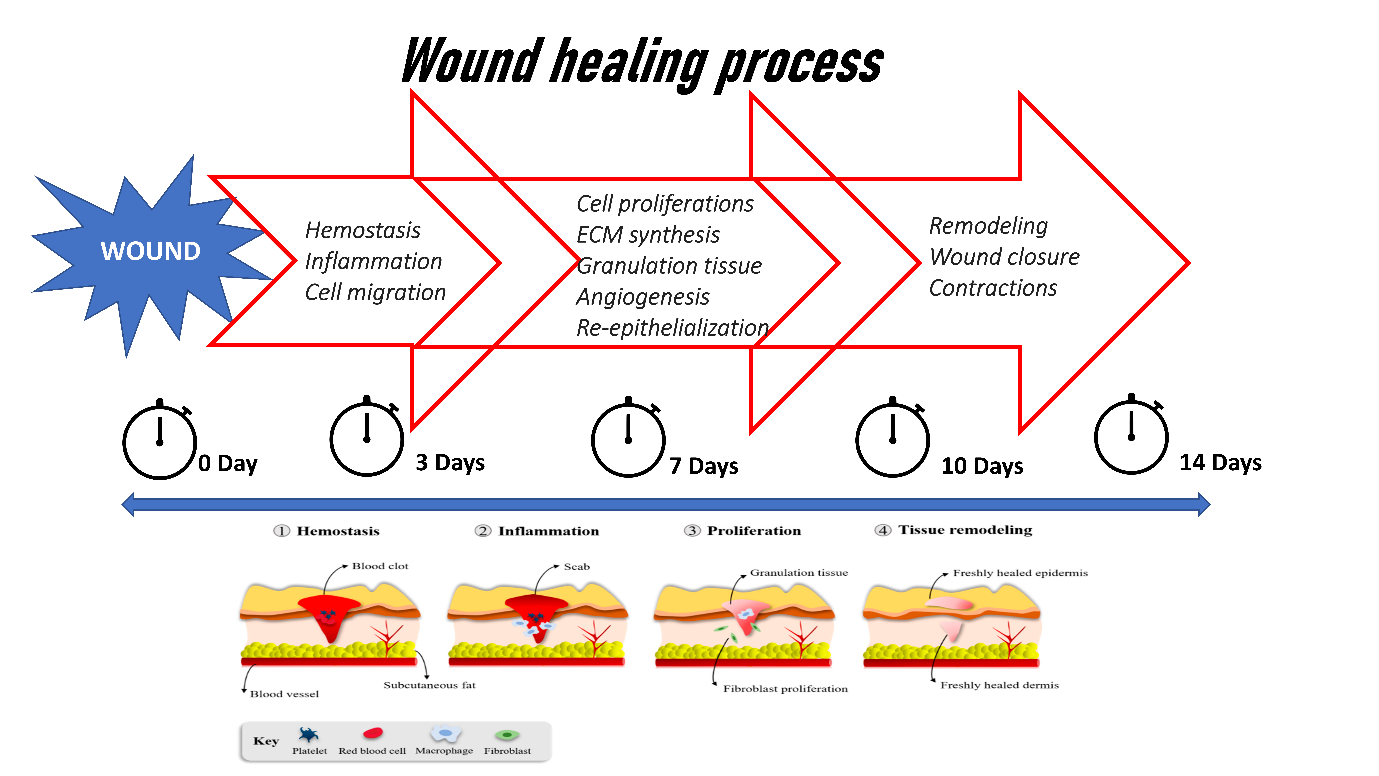
**Figure S1:** Different stages of wound healing [9]

Each of the skin layers plays a vital role in the wound healing process. The dermis is the layer of the skin that is located underneath the epidermis, and it is mainly composed of collagen and elastic fibers [13]. The subcutis is a layer of the dermis that is composed mostly of adipose tissue [14].

The function of these two layers is to protect deeper layers, provide a scaffold for new epithelial cells, and also supply crucial nutrients such as oxygen and nutrients that help with cell renewal [15,16].

Both layers play an important role in wound healing, but they have different functions. The outer layer takes care to resist shear force and stretch while providing selective permeability for water vapor, gasses, chemicals, and even bacteria [17,18]. The inner layer needs to provide space for epithelial cells to migrate outwards from the wound edges towards if fully healed state [19]. It also needs to provide a place for the cells to proliferate until they reach their full capacity [20].

Burn wounds are one of the most challenging damages and are among the most difficult to treat. Even though burn wounds immediately impact the skin, severe burns [(Total Body Surface Area) (TBSA) >20%] generate a systemic inflammatory response that damages the immune system, digestive system, and muscle throughout the body. This systemic damage is far more severe in burn injuries than in other types of trauma[21]. Understanding the pathophysiology of a burn injury is important for its effective management. The three recognized burn injury zones are coagulation, stasis, and hyperemia (Figure 2) [22]. The zone of coagulation is where irreversible coagulation of tissue proteins has occurred, and this region is consequently irretrievable. Decreased tissue perfusion is one of the major characteristics of the zone of stasis[23]. Hence, the primary objective of burn care is to increase blood flow to the affectoin order to avoid the spread of infection and serious damage. Therefore, the third zone of hyperemia is mostly out of danger unless there are other risk factors, such as the spreading of infection or very deep penetration of the wound.[24][25].

Key to the effective treatment of burn victims with varying degrees of skin damage is the prompt restoration of protective skin functions. Conventionally, autologous split or full-thickness skin grafts have been regarded as the finest final burn wound covering, but their use is restricted by the limited accessible supplies, particularly in cases of severe burns [26][27]. Donor site morbidities in the form of extra wounds and scars are also a risk of the autograft procedure[28].


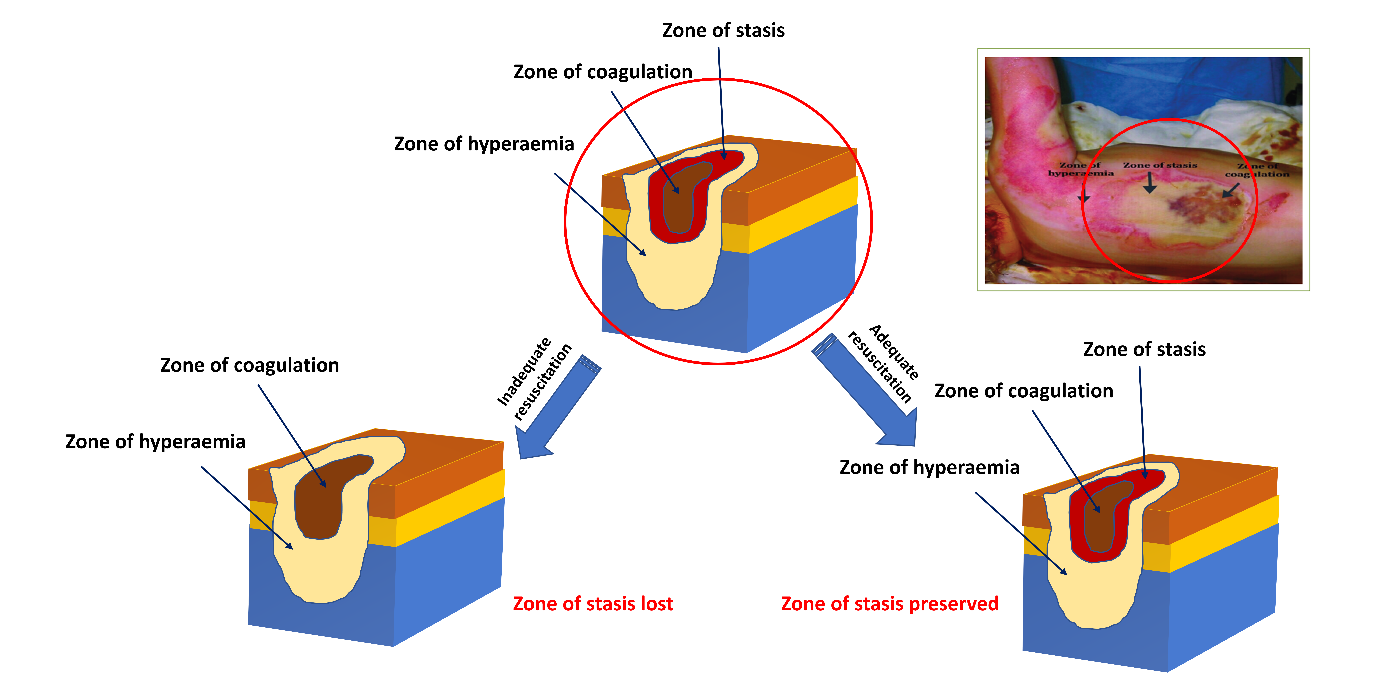


**Figure S2**: Schematic representation of burn injury zones in human skin[22]

The restoration rate of the skin on damaged areas is greatly reduced depending on the depth and severity of the injury leading to a slow healing process [29]. Several therapeutic practices have been utilized and are in continuous search to get effective wound healing methods with a higher successful scar healing rate, cost-effective practice, faster healing rate, and permanent remedies[30]. The regenerative approach is more popularized compared to any other technique as restoration of the damaged skin via re-establishment of deteriorated tissues is faster. This regenerative approach involves various techniques like tissue engineering, stem cell transplantation, biomaterials incorporation along with growth factors therapy[31–35].

Precisely the egression of skin tissue engineering is bestowed with the development of skin substitutes and other alternative bio-products using natural or synthetic materials for wound healing [36,37]. The process of tissue engineering includes several cells, and biomaterials, along with physiochemical and biochemical factors and sophisticated instrumentation for the replacement of damaged skin tissues [35].

Skin is inhabited by a different number of cells which are systematically arranged in a particular 3D structured matrix creating cell-matrix interaction. This micro-environment of typical skin structure is difficult to be artificially produced using conventional tissue engineering approaches [38]. Conventional tissue engineering majorly focuses on scaffold fabrication and cell seeding. In recent times conventional tissue engineering is facing a lot of hindrances, like lack of donors and transplant rejection, thus paving the way to some additional possible approaches serving as a complementary system. The choice of biomaterials also plays a major role in conventional tissue engineering. Consideration of Operative Specification, biocompatibility, and prior FDA approval have dominated material selection processes up to a great extent[39]. More, over compositional variation of bio-material, cell mimicking properties and its degradation rate also play a pivotal role in using 3D skin tissue engineering for the development of accelerated wound healing methods. In addition, with newer biomaterials being incorporated for fabrication as skin substitutes and/or use of stem cells for this purpose; the rate of wound healing, type of wound on which they are applied, cost-effectiveness, and stability of the constructed artificial structure under *in vivo* conditions should be given serious attention. [38].

The goal of burn wound care has changed from patient survival to getting better at improving the long-term shape and function of healed burn wounds and quality of life[40]. This change has led to the development of different skin substitutes for treating burn injuries.

Burn sufferers whose skin has been severely injured need to have their protective functions restored as quickly as possible[41]. Most burn wounds may be covered with autologous split or full-thickness skin transplants; however, this is restricted by the scarcity of donor skin for serious burns[42]. In such a situation, the only way is artificial grafting using different biomaterials.

An ideal burn wound skin substitute or graft should have the following properties[43]:

- Infectious pathogen resistance
- Prevents water from being lost
- Load Bearing Capacity
- Antigenicity is absent
- Flexible in terms of thickness
- Effective at a reasonable price
- Long-lasting and stable in the event of a wound.
- Maybe shaped to fit wounds with a variety of shapes and sizes.
- Biocompatible
- Readily accessible
- Long-lasting and simple to store.
- It's simple to secure and apply.

The last few years showed a great boost in the area of wound repair using bioactive materials. There is a huge spike in the use of some synthetic (PLGA, PVA, Polyurethanes, Silicone, PCL) and natural polymer-based(Gelatin, Starch, Chitosan, Cellulose) biomaterials for skin tissue engineering . The unique use of bioactive materials with genetic regulation of in situ tissue responses can both regenerate tissue and prevent tissue degeneration from occurring[44].

Biomaterials designed from different sources have proved highly advantageous for wound dressing and wound healing[45–47]. Many protein-based biomaterials, such as collagen and glycosaminoglycan-based agarose alginate, Gelatin, Chitin, Chitosan, and fibrin glues, have been shown to promote interactions with different cellular, owing to their intrinsic property[44]. This interaction leads to cell migration and attachment of cells to the biomaterials and hence leading to proper wound healing and scar management for burn wounds. Biomaterials from synthetic sources combined with natural polymers also showed promising results in wound management[47]. The Mechanical strength and extensive biocompatibility have made them suitable for wound care management[48]. Hydrogels/gels and gel-based composites, on the other hand, provide both repair and regeneration and thus can be considered to be optimal biomaterials for treating a burn[44]. Hydrogels and gels, owing to their unique structure, help in the digestion of necrotic and scar tissue while preventing excessive wound exudates from building up. Hydrogel also provides a cooling effect at the burn site[49,50]. The hydrogel can also be used in delivering various medications like hydrophobic antimicrobial drugs [51], Organic antibacterial agents like Tigecycline [52], tetracycline hydrochloride [53], inorganic antibacterial agents like Silver [54,55], Zinc[56,57] to the wound site and hence enhancing the wound healing rate[58,59].

Although several synthetic alternate fabrication methods of biomaterials insertion and regulation of wound healing are available yet, novel alternate techniques with higher efficiency, less cost, easy processing, and maximum success rate are always in search [60,61]. The combination of stem cells along with a novel biomaterial (Natural/synthetic) has provided higher impacts on the healing purpose of skin[62–64]. Any biomaterial could be an ideal one if cells can be incorporated within it easily and if it would accelerate the recovery of the damaged skin tissues for the healing process (Figure 3). Hence, this review highlights the different types of biomaterials used (Natural or synthetic) for improved treatment of different types of wound healing and dressing process. Moreover, the review further focuses on the most recent case studies of different biomaterials, their advantages, disadvantages, application, and outcome in various wound healing treatments.


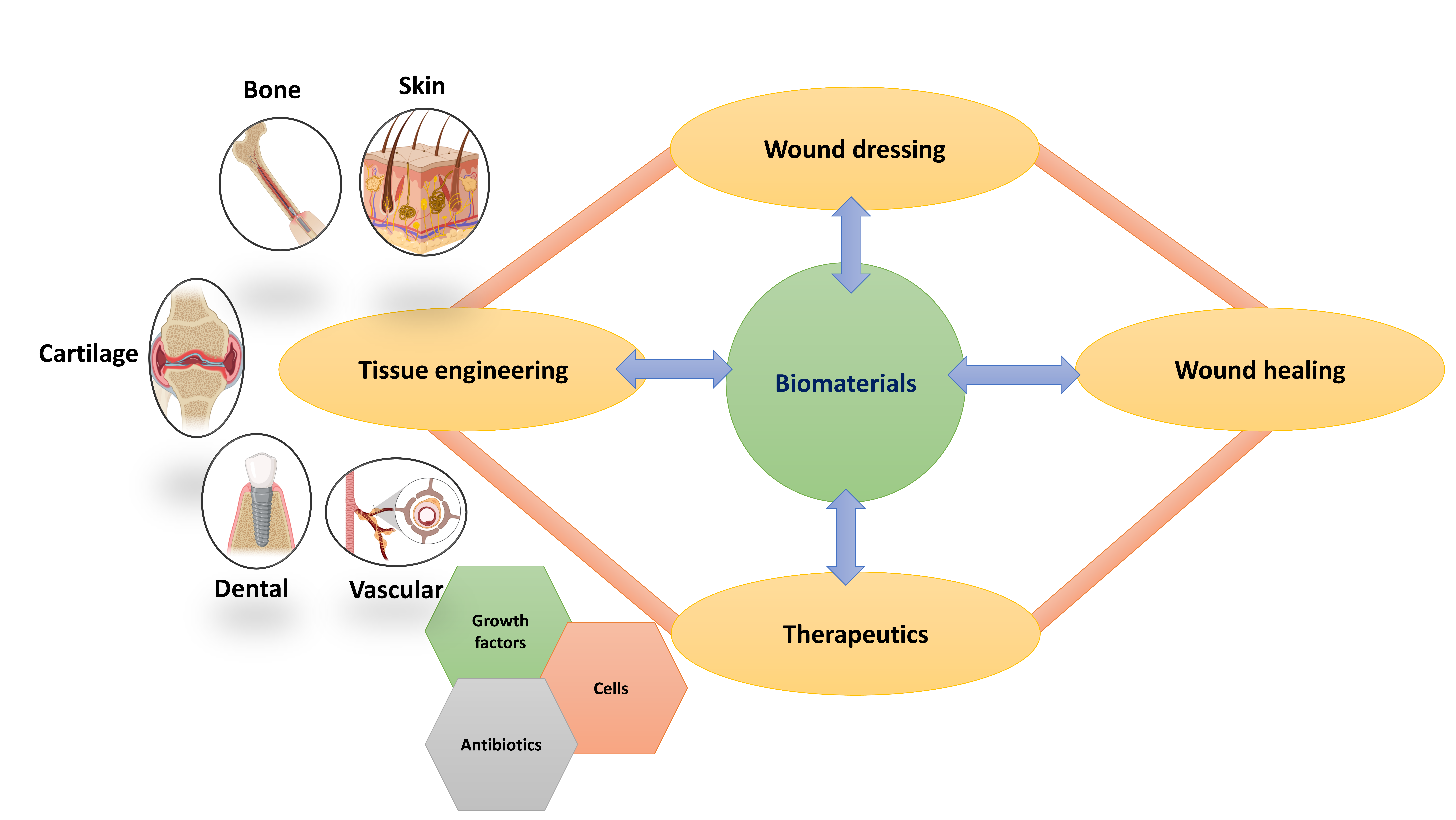
**Figure S3:** Multifunctional application of biomaterials with special emphasis on tissue engineering and wound healing

**References:**

1. Riha SM, Maarof M, Fauzi MB. Synergistic effect of biomaterial and stem cell for skin tissue engineering in cutaneous wound healing: A concise review. Polymers (Basel). 2021;13(10).

2. Kolarsick PAJ, Kolarsick MA, Goodwin C. Anatomy and physiology of the skin. J Dermatol Nurses Assoc. LWW; 2011;3(4):203–213.

3. Dutta P, Das S. Mammalian antimicrobial peptides: promising therapeutic targets against infection and chronic inflammation. Curr Top Med Chem. Bentham Science Publishers; 2016;16(1):99–129.

4. Mouritzen MV. Peptides and their effect on wound healing. Roskilde Universitet; 2019;

5. Liew PX, Kubes P. The neutrophil’s role during health and disease. Physiol Rev. American Physiological Society Bethesda, MD; 2019;99(2):1223–1248.

6. Zhang P, Sun B, Wu F, Zhang Q, Chu X, Ge M, et al. Wound healing acceleration by antibacterial biodegradable black phosphorus nanosheets loaded with cationic carbon dots. J Mater Sci. Springer; 2021;56(10):6411–6426.

7. Murray RZ, West ZE, Cowin AJ, Farrugia BL. Development and use of biomaterials as wound healing therapies. Burn Trauma. 2019;7.

8. Rezaie F, Momeni-Moghaddam M, Naderi-Meshkin H. Regeneration and Repair of Skin Wounds: Various Strategies for Treatment. Int. J. Low. Extrem. Wounds. 2019.(3).

9. Pinto AM, Cerqueira MA, Bañobre-Lópes M, Pastrana LM, Sillankorva S. Bacteriophages for chronic wound treatment: From traditional to novel delivery systems. Viruses. MDPI; 2020;12(2):235.

10. Ho J, Walsh C, Yue D, Dardik A, Cheema U. Current Advancements and Strategies in Tissue Engineering for Wound Healing: A Comprehensive Review. Adv. wound care. 2017.(6) p. 191–209.

11. Rognoni E, Watt FM. Skin Cell Heterogeneity in Development, Wound Healing, and Cancer. Trends Cell Biol. 2018.(9) p. 709–722.

12. Cai C, Meng Z, Zhao L, Wu T, Xu X, Zhu Y. A self-assembled peptide hydrogel for wound repair. J Mater Sci [Internet]. 2022; Available from: https://doi.org/10.1007/s10853-021-06607-5

13. Mohamed SA, Hargest R. Surgical anatomy of the skin. Surg. Elsevier; 2021;

14. Vestita M, Tedeschi P, Bonamonte D. Anatomy and Physiology of the Skin. Textb Plast Reconstr Surg. Springer; 2022. p. 3–13.

15. Lai-Cheong JE, McGrath JA. Structure and function of skin, hair and nails. Medicine (Baltimore). Elsevier; 2021;49(6):337–342.

16. Santoro D, Boyd M. The Skin as an Immune Organ. Diagnostics Ther Vet Dermatology. Wiley Online Library; 2021;1–8.

17. Dunnick CA, Gibran NS, Heimbach DM. Substance P has a role in neurogenic mediation of human burn wound healing. J Burn care \& Rehabil. Oxford University Press; 1996;17(5):390–396.

18. Yamaguchi Y, Yoshikawa K. Cutaneous wound healing: an update. J Dermatol. Wiley Online Library; 2001;28(10):521–534.

19. Rodrigues M, Kosaric N, Bonham CA, Gurtner GC. Wound healing: a cellular perspective. Physiol Rev. American Physiological Society Bethesda, MD; 2019;99(1):665–706.

20. Hausman DB, DiGirolamo M, Bartness TJ, Hausman GJ, Martin RJ. The biology of white adipocyte proliferation. Obes Rev. Wiley Online Library; 2001;2(4):239–254.

21. Tiwari VK. Burn wound: How it differs from other wounds? Indian J Plast Surg. Thieme Medical and Scientific Publishers Private Ltd.; 2012;45(02):364–373.

22. Hettiaratchy S, Dziewulski P. Pathophysiology and types of burns. Bmj. British Medical Journal Publishing Group; 2004;328(7453):1427–1429.

23. Singh V, Devgan L, Bhat S, Milner SM. The pathogenesis of burn wound conversion. Ann Plast Surg. LWW; 2007;59(1):109–115.

24. Yildirimer L, Thanh NTK, Seifalian AM. Skin regeneration scaffolds: a multimodal bottom-up approach. Trends Biotechnol. Elsevier; 2012;30(12):638–648.

25. Church D, Elsayed S, Reid O, Winston B, Lindsay R. Burn wound infections. Clin Microbiol Rev. Am Soc Microbiol; 2006;19(2):403–434.

26. Shakespeare P. Burn wound healing and skin substitutes. Burns. Elsevier; 2001;27(5):517–522.

27. Chen B, Liao Z, Guo Z, Huang X, Zhou Y. Burn wound care. Chinese Burn Surg. Springer; 2015. p. 113–169.

28. Mouarbes D, Dagneaux L, Olivier M, Lavoue V, Peque E, Berard E, et al. Lower donor-site morbidity using QT autografts for ACL reconstruction. Knee Surgery, Sport Traumatol Arthrosc. Springer; 2020;28(8):2558–2566.

29. Hesketh M, Sahin KB, West ZE, Murray RZ. Macrophage phenotypes regulate scar formation and chronic wound healing. Int. J. Mol. Sci. 2017.(7).

30. Yu P, Zhong W. Hemostatic materials in wound care. Burn Trauma [Internet]. 2021;9. Available from: https://doi.org/10.1093/burnst/tkab019

31. Hashemzadeh MR, Yazdi MET, Amiri MS, Mousavi SH. Stem cell therapy in the heart: Biomaterials as a key route. Tissue Cell. Elsevier; 2021;101504.

32. Mitsui R, Matsukawa M, Nakagawa K, Isomura E, Kuwahara T, Nii T, et al. Efficient cell transplantation combining injectable hydrogels with control release of growth factors. Regen Ther. Elsevier; 2021;18:372–383.

33. Liu T, Xu J, Pan X, Ding Z, Xie H, Wang X, et al. Advances of adipose-derived mesenchymal stem cells-based biomaterial scaffolds for oral and maxillofacial tissue engineering. Bioact Mater. Elsevier; 2021;6(8):2467–2478.

34. Nour S, Imani R, Chaudhry GR, Sharifi AM. Skin wound healing assisted by angiogenic targeted tissue engineering: A comprehensive review of bioengineered approaches. J Biomed Mater Res Part A. Wiley Online Library; 2021;109(4):453–478.

35. Tottoli EM, Dorati R, Genta I, Chiesa E, Pisani S, Conti B. Skin wound healing process and new emerging technologies for skin wound care and regeneration. Pharmaceutics. 2020.(8) p. 1–30.

36. Dye JF. From Secondary Intent to Accelerated Regenerative Healing: Emergence of the Bio-intelligent Scaffold Vasculogenic Strategy for Skin Reconstruction. Vasc Tissue Eng Regen Med. Springer; 2021;205–271.

37. Kanitkar A. Synthesis and Characterization of Novel Polyester Scaffolds from Sugarcane Industry By-products for Use in Skin and Bone Tissue Engineering. 2014;

38. Williams DF. Challenges With the Development of Biomaterials for Sustainable Tissue Engineering. Front Bioeng Biotechnol. 2019;7.

39. Adel IM, ElMeligy MF, Elkasabgy NA. Conventional and Recent Trends of Scaffolds Fabrication: A Superior Mode for Tissue Engineering. Pharmaceutics. MDPI; 2022;14(2):306.

40. Rowan MP, Cancio LC, Elster EA, Burmeister DM, Rose LF, Natesan S, et al. Burn wound healing and treatment: review and advancements. Crit care. Springer; 2015;19(1):1–12.

41. Halim AS, Khoo TL, Yussof SJM. Biologic and synthetic skin substitutes: an overview. Indian J Plast Surg. Thieme Medical and Scientific Publishers Private Ltd.; 2010;43(S 01):S23--S28.

42. Brusselaers N, Pirayesh A, Hoeksema H, Richters CD, Verbelen J, Beele H, et al. Skin replacement in burn wounds. J Trauma Acute Care Surg. LWW; 2010;68(2):490–501.

43. Shores JT, Gabriel A, Gupta S. Skin substitutes and alternatives: a review. Adv Ski \& wound care. LWW; 2007;20(9):493–508.

44. Ambrosio L. The role of biomaterials in burn treatment. Burn. \& trauma. BioMed Central; 2014.(4) p. 150–152.

45. Kumar SSD, Rajendran NK, Houreld NN, Abrahamse H. Recent advances on silver nanoparticle and biopolymer-based biomaterials for wound healing applications. Int J Biol Macromol. Elsevier; 2018;115:165–175.

46. Naomi R, Bahari H, Ridzuan PM, Othman F. Natural-based biomaterial for skin wound healing (Gelatin vs. collagen): Expert review. Polymers (Basel). MDPI; 2021;13(14):2319.

47. Mir M, Ali MN, Barakullah A, Gulzar A, Arshad M, Fatima S, et al. Synthetic polymeric biomaterials for wound healing: a review. Prog Biomater. Springer; 2018;7(1):1–21.

48. Mayet N, Choonara YE, Kumar P, Tomar LK, Tyagi C, Du Toit LC, et al. A comprehensive review of advanced biopolymeric wound healing systems. J Pharm Sci. Elsevier; 2014;103(8):2211–2230.

49. Stoica AE, Chircov C, Grumezescu AM. Hydrogel dressings for the treatment of burn wounds: an up-to-date overview. Materials (Basel). MDPI; 2020;13(12):2853.

50. Jandera V, Hudson DA, De Wet PM, Innes PM, Rode H. Cooling the burn wound: evaluation of different modalites. Burns. Elsevier; 2000;26(3):265–270.

51. Fang K, Wang R, Zhang H, Zhou L, Xu T, Xiao Y, et al. Mechano-responsive, tough, and antibacterial zwitterionic hydrogels with controllable drug release for wound healing applications. ACS Appl Mater \& Interfaces. ACS Publications; 2020;12(47):52307–52318.

52. Nimal TR, Baranwal G, Bavya MC, Biswas R, Jayakumar R. Anti-staphylococcal activity of injectable nano tigecycline/chitosan-PRP composite hydrogel using Drosophila melanogaster model for infectious wounds. ACS Appl Mater \& interfaces. ACS Publications; 2016;8(34):22074–22083.

53. Anjum S, Arora A, Alam MS, Gupta B. Development of antimicrobial and scar preventive chitosan hydrogel wound dressings. Int J Pharm. Elsevier; 2016;508(1–2):92–101.

54. Martins AF, Monteiro JP, Bonafe EG, Gerola AP, Silva CTP, Girotto EM, et al. Bactericidal activity of hydrogel beads based on N, N, N-trimethyl chitosan/alginate complexes loaded with silver nanoparticles. Chinese Chem Lett. Elsevier; 2015;26(9):1129–1132.

55. Jaiswal M, Koul V, Dinda AK. In vitro and in vivo investigational studies of a nanocomposite-hydrogel-based dressing with a silver-coated chitosan wafer for full-thickness skin wounds. J Appl Polym Sci. Wiley Online Library; 2016;133(21).

56. Kumar PS, Lakshmanan VK, Anilkumar T. C, Ramya, P. Reshmi, A. Unnikrishnan. Flexible and microporus chitosan hydrogel/nano-ZnO composite bandages for wound dressing: in vitro and in vivo evaluation, Appl. Mater. Interfaces (Providence). 2012;4:2618–2629.

57. Nair S, Sasidharan A, Divya Rani V V, Menon D, Nair S, Manzoor K, et al. Role of size scale of ZnO nanoparticles and microparticles on toxicity toward bacteria and osteoblast cancer cells. J Mater Sci Mater Med. Springer; 2009;20(1):235–241.

58. Liu H, Wang C, Li C, Qin Y, Wang Z, Yang F, et al. A functional chitosan-based hydrogel as a wound dressing and drug delivery system in the treatment of wound healing. RSC Adv. Royal Society of Chemistry; 2018;8(14):7533–7549.

59. Boateng JS, Matthews KH, Stevens HNE, Eccleston GM. Wound healing dressings and drug delivery systems: a review. J Pharm Sci. Elsevier; 2008;97(8):2892–2923.

60. Cendere S. Design and characterization of hydrogel models containing NK-92 or HEK293T cells for skin tissue engineering. Ac{\i}badem Mehmet Ali Ayd{\i}nlar Üniversitesi, Sa{\u{g}}l{\i}k Bilimleri Enstitüsü; 2021.

61. Tang SLP. Dressings for Advanced Wound Care. CRC Press; 2021.

62. Wei C, Feng Y, Che D, Zhang J, Zhou X, Shi Y, et al. Biomaterials in skin tissue engineering. Int J Polym Mater Polym Biomater. Taylor \& Francis; 2021;1–19.

63. Sevari SP, Ansari S, Moshaverinia A. A narrative overview of utilizing biomaterials to recapitulate the salient regenerative features of dental-derived mesenchymal stem cells. Int J Oral Sci. Nature Publishing Group; 2021;13(1):1–12.

64. Kong F, Mehwish N, Niu X, Lin M, Rong X, Hu F, et al. Personalized hydrogels for individual health care: Preparation, features, and applications in tissue engineering. Mater Today Chem. Elsevier; 2021;22:100612.
